# N^6^‐Methyladenosine‐Modified CBX1 Regulates Nasopharyngeal Carcinoma Progression Through Heterochromatin Formation and STAT1 Activation

**DOI:** 10.1002/advs.202205091

**Published:** 2022-10-30

**Authors:** Yin Zhao, Shengyan Huang, Xirong Tan, Liufen Long, Qingmei He, Xiaoyu Liang, Jiewen Bai, Qingjie Li, Jiayi Lin, Yingqin Li, Na Liu, Jun Ma, Yupei Chen

**Affiliations:** ^1^ Sun Yat‐sen University Cancer Center State Key Laboratory of Oncology in South China Collaborative Innovation Center of Cancer Medicine Guangdong Key Laboratory of Nasopharyngeal Carcinoma Diagnosis and Therapy 651 Dongfeng Road East Guangzhou Guangdong 510060 China

**Keywords:** CBX1, heterochromatin, m^6^A, MAP7, nasopharyngeal carcinoma, PD‐L1

## Abstract

Epitranscriptomic remodeling such as N^6^‐methyladenosine (m^6^A) modification plays a critical role in tumor development. However, little is known about the underlying mechanisms connecting m^6^A modification and nasopharyngeal carcinoma (NPC) progression. Here, CBX1 is identified, a histone methylation regulator, to be significantly upregulated with m^6^A hypomethylation in metastatic NPC tissues. The m^6^A‐modified CBX1 mRNA transcript is recognized and destabilized by the m^6^A reader YTHDF3. Furthermore, it is revealed that CBX1 promotes NPC cell migration, invasion, and proliferation through transcriptional repression of MAP7 via H3K9me3‐mediated heterochromatin formation. In addition to its oncogenic effect, CBX1 can facilitate immune evasion through IFN‐*γ*‐STAT1 signaling‐mediated PD‐L1 upregulation. Clinically, CBX1 serves as an independent predictor for unfavorable prognosis in NPC patients. The results reveal a crosstalk between epitranscriptomic and epigenetic regulation in NPC progression, and shed light on the functions of CBX1 in tumorigenesis and immunomodulation, which may provide an appealing therapeutic target in NPC.

## Graphical abstract

m^6^A methylomes are mapping in nasopharyngeal carcinoma (NPC) using m^6^A sequencing, and CBX1 is identified to be significantly upregulated with m^6^A hypomethylation in metastatic NPC tissues. The functions of CBX1 in promoting NPC tumorigenesis and immune evasion are further uncovered, in which CBX1 facilitates transcriptional repression of MAP7 via H3K9me3‐mediated heterochromatin formation and activates IFN‐*γ*‐STAT1 signaling‐mediated PD‐L1 upregulation.

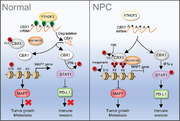

## 1 Introduction

Nasopharyngeal carcinoma (NPC), which arises from the nasopharyngeal pharynx, is a metastasis‐prone malignancy highly prevalent in East and Southeast Asia, especially southern China.^[^ ^1^ ^]^ Radiotherapy in combination with chemotherapy is the primary therapy for NPC.^[^ ^2^ ^]^ However, despite progress in chemo‐radiotherapeutic strategies and new systemic treatments, such as programmed death­1 (PD‐1) blockade, ≈20% of NPC patients still suffer from distant metastasis, and the response rate to PD‐1 inhibitors is generally low (≈20–30%).^[^ ^3^ ^]^ Thus, addressing the regulatory mechanisms underlying NPC progression to develop new therapeutic strategies for this disease is of vital importance.

Genetically, NPC shows relatively few recurrent somatic point mutations or copy number alterations.^[^ ^4^ ^]^ In contrast, epigenetic aberrations (e.g., DNA methylation) have been frequently reported in NPC.^[^ ^5^ ^]^ Histone methylation is a critical determinant of chromatin states and a key marker of transcriptional gene regulation.^[^ ^6^ ^]^ This modification can be dynamically regulated by histone methyltransferases (writers), demethylases (erasers), and methylation reader proteins. Recent studies have shown that alterations in histone methylation are closely involved in NPC progression. For example, the histone methyltransferase EZH2 represses IKK*α* expression via H3K27 histone methylation and impairs NPC differentiation.^[^ ^7^ ^]^ Silencing of KDM4A, a histone demethylase, can inhibit NPC migration and invasion.^[^ ^8^ ^]^ However, the mechanisms involving these histone methylation regulators in NPC progression are largely unknown.

More recently, N^6^‐methyladenosine (m^6^A) RNA modification, the most abundant epitranscriptomic modification in eukaryotic mRNA, has attracted great attention in cancer research because of its vital biological functions.^[^ ^9^ ^]^ This reversible modification is catalyzed by m^6^A modifiers including methyltransferase complexes (e.g., METTL3 and METTL14) and demethylases (e.g., ALKBH5 and FTO), and the fate of m^6^A‐modified mRNA is ultimately determined by different reader proteins (e.g., YTHDFs and YTHDCs).^[^ ^10^ ^]^ Although dysregulation of the m^6^A modification has been studied extensively in tumor pathogenesis，^[^ ^11^ ^]^ its importance in the development and progression of NPC remains elusive. Recently, it was shown that METTL3‐ and WTAP‐mediated m^6^A enrichment can facilitate NPC growth and metastasis.^[^ ^12^ ^]^ He et al. reported that YTHDC2 could enhance the translation initiation of IGF1R mRNA to promote radiotherapy resistance in NPC cells.^[^ ^13^ ^]^ Although m^6^A alters mRNA stability and translation, rapidly accumulating evidence highlights the significance of crosstalk between this epitranscriptomic modification and histone epigenetic regulation in physio‐pathological cellular processes.^[^ ^14^ ^]^ Whether histone methylation modulators can be regulated by m^6^A modification and, if so, what they do and how they act in NPC development and progression remain to be elucidated.

Here, beginning with the hypothesized existence of an interaction between m^6^A and histone methylation regulators, we analyzed the m^6^A modification profile in paired NPC tissues and identified that CBX1 (also called HP1‐*β*), a histone methylation reader protein, was highly expressed with m^6^A hypomethylation in metastatic NPC. Specifically, we found that the m^6^A‐modified CBX1 mRNA transcript was recognized and destabilized by the m^6^A reader YTHDF3. Furthermore, we demonstrated that CBX1 could promote NPC cell migration, invasion and proliferation in vitro and in vivo through heterochromatin formation‐mediated inhibition of MAP7, indicating crosstalk between epitranscriptomic and epigenetic regulation in NPC tumorigenesis. In addition to its oncogenic effect, we observed a correlation between high CBX1 expression and an impaired anti‐PD‐1 response, and found that CBX1 could inhibit the NPC‐cell killing by chimeric antigen receptor (CAR)‐T cells in vitro through PD‐L1 upregulation mediated by IFN‐*γ*‐STAT1 signaling. Clinically, CBX1 was identified as an independent predictor for unfavorable prognosis in NPC patients. Our results uncover the roles of CBX1 in NPC tumorigenesis and immunomodulation and indicate that CBX1 may serve as an appealing therapeutic target in NPC.

## 2 Results

### 2.1 Identification of CBX1 as an m^6^A‐Modified Histone Methylation Regulator in NPC

**Figure 1:**
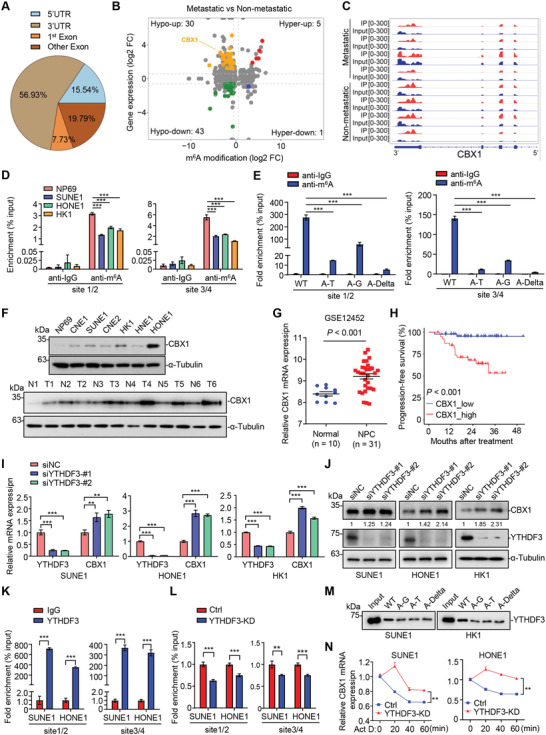
Identification of CBX1 as an m^6^A‐modified histone methylation regulator in NPC. A,B) m^6^A‐seq analysis in NPC biopsy tissue samples with (*n* = 4) or without (*n* = 4) metastasis. Distribution of genes with significantly altered m^6^A levels (|fold change| ≥ 1.5 and *P* < 0.05) and expression levels (|fold change| ≥ 1.5 and *P* < 0.05). C) m^6^A peaks were enriched in 3′UTR of CBX1 gene from m^6^A‐seq data. D) m^6^A‐RIP‐qPCR analysis of CBX1 m^6^A levels in NP69, SUNE1, HONE1, and HK1 cells. Mean (*n* = 3) ± s.d. One‐way ANOVA, *** *P* < 0.001. E) m^6^A‐RIP‐qPCR analysis of CBX1 m^6^A levels in HK1 cells transfected with WT, A‐T, A‐G, or A‐Delta CBX1 mRNA fragment constructs. Mean (*n* = 3) ± s.d. One‐way ANOVA, *** *P* < 0.001. F) Western blotting of CBX1 and *α*‐Tubulin in NP69, CNE1, CNE2, HK1, SUNE1, HONE1, and HNE1 cells. G) Relative mRNA levels of CBX1 in normal (*n* = 10) and NPC (*n* = 31) tissue samples in the GSE12452 dataset. H) Kaplan–Meier progression‐free survival rates for NPC patients having high CBX1 (red) or low CBX1 (blue) expression in the GSE102349 dataset. Log‐rank test. I) Relative mRNA levels of CBX1 and YTHDF3 in SUNE1, HONE1 or HK1 cells transfected with siRNA targeting NC (siNC) or YTHDF3 (siYTHDF3‐#1 or ‐#2). Mean (*n* = 3) ± s.d. One‐way ANOVA, ** *P* < 0.01, *** *P* < 0.001. J) Western blotting of CBX1, YTHDF3 and *α*‐Tubulin in SUNE1, HONE1 or HK1 cells transfected with siRNA targeting NC (siNC) or YTHDF3 (siYTHDF3‐#1 or ‐#2). K) RIP‐qPCR analysis of IgG and YTHDF3 occupation in CBX1 m^6^A sites in SUNE1 or HONE1 cells. Mean (*n* = 3) ± s.d. Student's *t*‐test, *** *P* < 0.001. L) RIP‐qPCR analysis of YTHDF3 occupation in CBX1 m^6^A sites in SUNE1 or HONE1 cells transfected with siRNA targeting NC (Ctrl) or YTHDF3 (YTHDF3‐KD). Mean (*n* = 3) ± s.d. Student's *t*‐test, *** *P* < 0.001. M) Western blotting of the interaction of YTHDF3 with WT, A‐G, A‐T or A‐Delta CBX1 mRNA fragments from RNA pulldown assays using SUNE1 or HK1 cell lysates. N) Relative mRNA levels of CBX1 in SUNE1 or HONE1 cells transfected with siRNA targeting NC (siNC) or YTHDF3 (siYTHDF3‐KD) followed by treatment with actinomycin D (1 µg mL^−1^) by indicated time. Mean (*n* = 3) ± s.d. Two‐way ANOVA, ** *P* < 0.01.

We started by investigating the variations in m^6^A modification in NPC by mapping m^6^A methylomes in four paired NPC tissue samples with or without metastasis by m^6^A‐RIP sequencing (m^6^A‐seq) (Figure S1A, Supporting Information). The results showed that the m^6^A peaks were mainly enriched in the 3′ untranslated region (UTR) in NPC, which was consistent with previous reports (**Figure** **1**A).^[^ ^9^, ^10^ ^]^ Further m^6^A‐seq analysis distinguished 2792 differential m^6^A peaks (diff‐peaks; ≥1.5‐fold change and *P* < 0.05) between NPC tissues with or without metastasis, and a total of 79 diff‐peaks corresponding to 70 genes were identified when the m^6^A‐seq data were combined with differentially expressed genes identified by RNA sequencing (RNA‐seq) analysis (≥1.5‐fold change and *P* < 0.05) (Figure S1A, Supporting Information). Our previous studies revealed that epigenetic alterations, such as gene silencing resulting from DNA hypermethylation, are involved in NPC progression.^[^ ^15^ ^]^ However, despite histone methylation regulators being important players in epigenetic mechanisms, their roles in NPC remain poorly understood. Considering the potential crosstalk between epitranscriptomic and epigenetic regulation, we speculated that histone methylation regulators may be regulated by m^6^A modification during NPC progression. To test this idea, we analyzed the 187 writers, erasers and readers involved in histone methylation, which were identified from the WERAM database (http://weram.biocuckoo.org/)^[^ ^16^ ^]^ (Table S1, Supporting Information), with the abovementioned 79 diff‐peaks and identified that CBX1, a histone methylation reader, was the only gene with a significant diff‐peak in the hypo‐up group (Figure 1B; Figure S1A, Supporting Information).

The m^6^A‐seq analysis showed that the m^6^A peaks were mainly enriched in the 3′UTR of CBX1 (Figure 1C). Nine “A” sites in accordance with the “RRACH” principal were identified within this 3′UTR (data not shown). To further verify the exact m^6^A sites of CBX1, we evaluated anti‐m^6^A antibody‐enriched CBX1 mRNA from normal nasopharyngeal epithelial cells (NP69) and NPC cells (SUNE1, HONE1, and HK1) with m^6^A‐RIP‐qPCR assays using three different primers, the products of which contained the nine “A” sites. The results showed that two of them could amplify the indicated fragments containing the 1/2 and 3/4 “A” sites, indicating that at least two of these four “A” sites are m^6^A‐modified, and the m^6^A levels of CBX1 mRNA in SUNE1, HONE1 and HK1 cells were significantly lower than those in NP69 cells (Figure 1D; Figure S1B, Supporting Information). To further confirm these results, we constructed plasmids encoding mRNA fragments of CBX1 wild type (WT) and three mutants (four Alanine to Glycine, four Alanine to Threonine or four Alanine to deletion, named A‐G, A‐T, and A‐Delta, respectively), and then transfected these constructs into HK1 cells. The total RNA was extracted for further m^6^A‐RIP‐qPCR analysis. The results showed that cells overexpressing the three mutants had significantly lower m^6^A levels of CBX1 mRNA compared to those overexpressing the WT construct, supporting that CBX1 was m^6^A‐modified on the fragment containing those four “A” sites in NPC cells (Figure 1E). Then, we investigated the protein expression of CBX1 and found that it was remarkably higher in NPC cell lines and tissues than in normal controls (Figure 1F). In addition, by analyzing the public GSE12452 and GSE102349 datasets, the mRNA levels of CBX1 in NPC tissues were found to be significantly higher than those in normal tissues, while high mRNA expression of CBX1 was associated with poor progression‐free survival (PFS) in NPC (Figure 1G,H).

Next, to verify whether m^6^A methylation of CBX1 mRNA affects CBX1 mRNA expression in NPC, we first analyzed the correlations between the mRNA expression levels of *CBX1* and common m^6^A regulators using the transcriptome data for NPC cells at single‐cell resolution from our previous study (GSE150430).^[^ ^17^ ^]^ Notably, the expression of members of the YTH domain family showed relatively high correlations with that of CBX1 (Figure S1C, Supporting Information). We then constructed shRNAs or siRNAs targeting the YTHDF reader family and investigated the effects of member knockdown on the expression of CBX1 in NPC cells. The results showed that knockdown of YTHDF3 significantly promoted the mRNA expression of CBX1, while knockdown of YTHDF1 or YTHDF2 had little effect (Figure 1I; Figure S1D,E, Supporting Information). We further confirmed that knocking down YTHDF3 increased the protein levels of CBX1 in NPC cells (Figure 1J). These data suggest that YTHDF3 can regulate the mRNA and protein expression levels of CBX1 in NPC cells.

YTHDF3 is an m^6^A reader that can regulate mRNA decay or translation.^[^ ^18^ ^]^ However, its role in NPC remains unknown. We then evaluated the mRNA expression of YTHDF3 in NPC and found that YTHDF3 was downregulated in NPC cells compared to normal nasopharyngeal epithelial cells (Figure S1F, Supporting Information). In addition, the mRNA expression level of YTHDF3 was lower in NPC tissues than in normal tissues, and lower expression of YTHDF3 was associated with poorer PFS in public NPC datasets (Figure S1G,H, Supporting Information). Furthermore, IHC staining showed significantly negative correlation between CBX1 and YTHDF3 expression in NPC tissues (Figure S1I, Supporting Information). To further investigate whether YHTDF3 can directly recognize m^6^A modification of CBX1 mRNA, we performed RNA immunoprecipitation (RIP) assays with an anti‐YTHDF3 antibody in NPC cells (Figure S1J, Supporting Information). The results showed that the CBX1 mRNA in NPC cells was remarkably enriched by the anti‐YTHDF3 antibody (Figure 1K), but this relative enrichment was significantly suppressed in YTHDF3‐knockdown cells (Figure 1L; Figure S1K, Supporting Information). In addition, RNA pulldown assays showed that YTHDF3 protein pulled down by the mutants (A‐G, A‐T, or A‐Delta) was much less than those by WT CBX1 fragment (Figure 1M). Furthermore, knocking down YTHDF3 significantly decreased the decay of CBX1 mRNA induced by treatment with the transcription inhibitor actinomycin D (Act‐D) (Figure 1N; Figure S1L, Supporting Information). Collectively, these data suggest that the histone methylation reader CBX1 is highly expressed with m^6^A hypomethylation in NPC and that the m^6^A‐modified transcript of CBX1 can be recognized and destabilized by YTHDF3.

### 2.2 CBX1 Inhibition Suppresses NPC Cell Migration, Invasion, and Proliferation and Enhances the NPC‐Cell Killing by CAR‐T Cells in Vitro

CBX1 belongs to the heterochromatin protein 1 (HP1) family, which includes the three members HP1‐*α* (CBX5), HP1‐*β* (CBX1) and HP1‐*γ* (CBX3). It has been reported that CBX1 regulates the biological processes of chromosome segregation, DNA repair and RNA splicing and plays an important role in epigenetic gene silencing.^[^ ^19^ ^]^ However, the function and clinical relevance of CBX1 in NPC have not yet been investigated.

**Figure 2:**
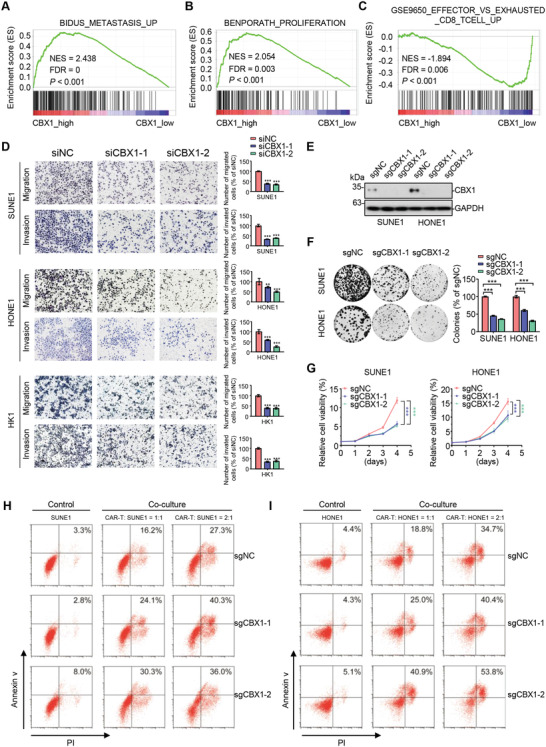
CBX1 inhibition suppresses NPC cell migration, invasion and proliferation and enhances the NPC‐cell killing by CAR‐T cells in vitro. A–C) GSEA analysis based on GSE102349 dataset showing gene sets related to metastasis A), proliferation B), and immune evasion C) enriched in CBX1‐high NPC tissues. D) Representative images and quantification of migration and invasion transwell assays in SUNE1, HONE1 or HK1 cells transfected with siRNA targeting NC (siNC) or CBX1 (siCBX1‐1 or ‐2). Mean (*n* = 5) ± s.d. One‐way ANOVA, ** *P* < 0.01, *** *P* < 0.001. E) Western blotting of CBX1 and GAPDH in SUNE1 and HONE1 cells stably transfected with sgRNA targeting NC (sgNC) or CBX1 (sgCBX1‐1 or ‐2). F) Representative images and quantification of colony formation assays in SUNE1 and HONE1 cells stably transfected with sgRNA targeting NC (sgNC) or CBX1 (sgCBX1‐1 or ‐2). Mean (*n* = 3) ± s.d. One‐way ANOVA, *** *P* < 0.001. G) CCK‐8 assays in SUNE1 and HONE1 cells stably transfected with sgRNA targeting NC (sgNC) or CBX1 (sgCBX1‐1 or ‐2). Mean (*n* = 6) ± s.d. One‐way ANOVA, *** *P* < 0.001. H,I) The CAR‐T cells were added at 0:1, 1:1 or 2:1 ratio to sgNC, sgCBX1‐1, or sgCBX1‐2 and incubated for 24 h at 37 °C. The whole cells were collected for Annexin‐V/PI staining and for further flow cytometric analyzing.

To explore the functional role of CBX1 in NPC, we performed gene set enrichment analysis (GSEA) to compare the gene profiles of NPC samples with high and low CBX1 expression using the GSE102349 dataset. We found that the gene sets related to metastasis, proliferation, and the immune response were strongly enriched in CBX1‐high NPC samples (**Figure** **2**A–C; Figure S2A–C, Supporting Information). We then confirmed that overexpression of CBX1 remarkably promoted cell migration and proliferation in HK1 cells (Figure S2D–G, Supporting Information). Conversely, knockdown of CBX1 inhibited NPC cell migration, invasion and proliferation (Figure 2D; Figure S2H–K, Supporting Information). To further confirm the oncogenic role of CBX1 in NPC, we generated CBX1‐knockout cells through CRISPR/Cas9‐mediated gene transfer (Figure 2E; Figure S2L, Supporting Information) and repeated the functional studies with NPC cells. We found that knockout of CBX1 also inhibited NPC cell proliferation in vitro (Figure 2E–G). In addition to the oncogenic effect, significant enhancement of NPC‐cell killing by CAR‐T cells was observed; this effect was dependent on HER2 expression on the NPC cell surface, and knocking out CBX1 did not reduce HER2 expression (Figure 2H,I; Figure S2M, Supporting Information). Consistent with these observations, we found that CBX1 expression in NPC cells was positively correlated with the proportion of exhausted CD8^+^ T cells by analyzing our single‐cell RNA‐seq data reported previously (GSE150430, Figure S2N, Supporting Information).^[^ ^17^ ^]^ These results demonstrated that targeting CBX1 could inhibit NPC cell migration, invasion and proliferation and enhance the antitumor immune response in NPC.

### 2.3 CBX1 Inhibition Impairs NPC Tumorigenesis In Vivo

**Figure 3:**
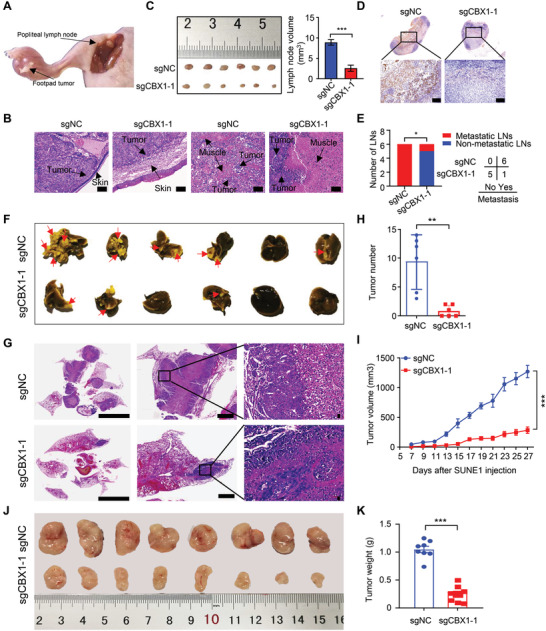
CBX1 inhibition impairs NPC tumorigenesis in vivo. A‐E) SUNE1 cells stably transfected with sgRNA targeting NC (sgNC) or CBX1 (sgCBX1‐1) were injected into footpads of nude mice to construct an inguinal lymph node metastasis model. Representative images of primary foot pad tumor and metastatic inguinal lymph node A) and the primary tumors in footpads following H&E staining B); images and quantification of the volumes of the inguinal lymph nodes C); representative images of IHC staining (anti‐keratin) of the inguinal lymph nodes D); number of metastasis and nonmetastasis inguinal lymph nodes E). Mean (*n* = 6) ± s.d. Student's *t*‐test in (C), chi‐square (*χ* ^2^) test in (E), * *P* < 0.05, *** *P* < 0.001. Scale bars, 100 µm. F‐H) SUNE1 cells stably transfected with sgRNA targeting NC (sgNC) or CBX1 (sgCBX1‐1) were injected into the tail vein of nude mice to construct a lung metastasis model. Images of lungs F), representative images of H&E staining of lungs G), and quantification of metastatic nodules in lungs H). Mean (*n* = 6) ± s.d. Student's *t*‐test, ** *P* < 0.01. Scale bars, 6 mm. I–K) SUNE1 cells stably transfected with sgRNA targeting NC (sgNC) or CBX1 (sgCBX1‐1) were injected into the axilla of nude mice to construct tumor growth model. Tumor growth curves I), images J), and weight K) of tumors. Mean (*n* = 8) ± s.d. Two‐way ANOVA, *** *P* < 0.001.

To further characterize the oncogenic function of CBX1 in vivo, an inguinal lymph node metastasis model, lung metastasis model and subcutaneous xenograft model established in nude mice were employed. We found that knockout of CBX1 suppressed the invasion of NPC cells into the plantae skin and muscles (**Figure** **3**A,B). In addition, knockout of CBX1 suppressed the enlargement of the ipsilateral inguinal lymph node and the metastasis of NPC cells to the ipsilateral inguinal lymph node (Figure 3C–E). Consistently, knocking out CBX1 remarkably suppressed lung metastasis by NPC cells (Figure 3F–H). The above results indicated that knockout of CBX1 inhibited the invasion and metastasis of NPC cells in vivo. Furthermore, the results from the subcutaneous xenograft model showed that knocking out CBX1 significantly inhibited tumor growth (Figure 3I–K) and that tumor tissues in the CBX1‐knockout group had lower expression of Ki67 and PCNA than those in the sgNC group (Figure S3A,B, Supporting Information). Besides, we generated sgNC+Vec, sgCBX1+Vec and sgCBX1+CBX1 cell lines by reconstructing empty vector or CBX1 into sgNC or sgCBX1 SUNE1 cells (Figure S4A, Supporting Information), and established the lymph nodes metastasis and subcutaneous xenograft mouse model. We found that restoring the expression of CBX1 significantly reversed the inhibitory effect of CBX1 knockout on NPC cell metastasis and tumor growth (Figure S4B‐G, Supporting Information). Taken together, these data demonstrate that CBX1 promotes NPC tumorigenesis in vivo.

### 2.4 CBX1 Transcriptionally Represses MAP7 via H3K9me3‐Mediated Heterochromatin Formation

**Figure 4:**
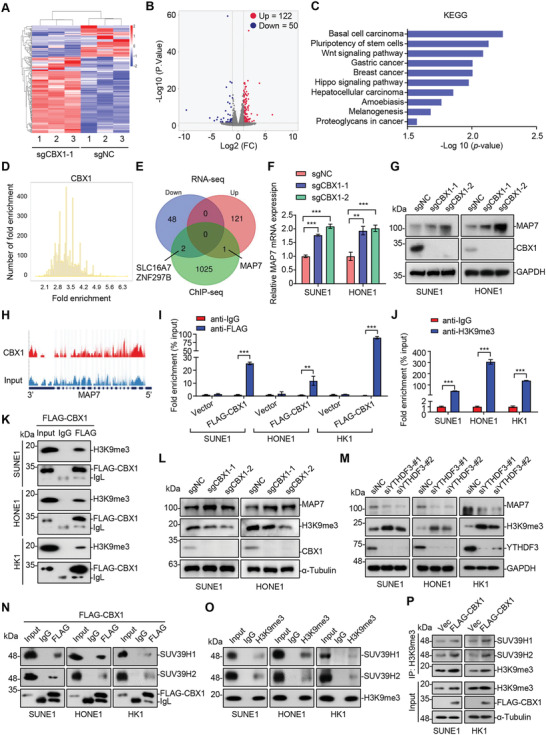
CBX1 transcriptionally represses MAP7 via H3K9me3‐mediated heterochromatin formation. A,B) Heatmap A) and volcanic map B) showing differentially expressed genes (|fold change| ≥ 2 and *q* value < 0.05) of RNA‐seq analysis in SUNE1 cells stably transfected with sgRNA targeting NC (sgNC) or CBX1 (sgCBX1‐1). Data are presented as three technical replications. C) KEGG analysis of differential genes through RNA‐seq upon CBX1 knockout. D) ChIP‐seq analysis of genes that enriched by CBX1 in SUNE1 cells with CBX1 overexpression (*q* value < 0.05). E) Venn diagram showing the overlap genes between RNA‐seq (|fold change| ≥ 2 and *q* value < 0.05) and ChIP‐seq (*q* value < 0.05) peaks. F,G) Relative mRNA levels of MAP7 F) and western blotting of MAP7, CBX1, and GAPDH G) in SUNE1 and HONE1 cells stably transfected with sgRNA targeting NC (sgNC) or CBX1 (sgCBX1‐1 or ‐2). Mean (*n* = 3) ± s.d. One‐way ANOVA, ** *P* < 0.01, *** *P* < 0.001. H) ChIP‐seq traces of MAP7 signal in SUNE1 cells that overexpressed FLAG‐CBX1. I) qPCR analysis of CBX1 occupation on MAP7 binding sites following by ChIP experiment (anti‐IgG or anti‐FLAG) in SUNE1, HONE1 or HK1 cells expressing empty vector or FLAG‐CBX1. Mean (*n* = 3) ± s.d. Two‐way ANOVA, *** *P* < 0.001. J) qPCR analysis of H3K9me3 occupation on MAP7 binding sites following by ChIP experiment (anti‐IgG or anti‐H3K9me3) in SUNE1, HONE1 or HK1 cells. Mean (*n* = 3) ± s.d. Student's *t*‐test, *** *P* < 0.001. K) Immunoprecipitation (with anti‐IgG or anti‐FLAG) and western blotting of H3K9me3 and FLAG‐CBX1 in SUNE1, HONE1, or HK1 cells expressing FLAG‐CBX1. L) Western blotting of MAP7, H3K9me3, CBX1, and *α*‐Tubulin in SUNE1 and HONE1 cells stably transfected with sgRNA targeting NC (sgNC) or CBX1 (sgCBX1‐1 or ‐2). M) Western blotting of MAP7, H3K9me3, YTHDF3, and GAPDH in SUNE1, HONE1, or HK1 cells transfected with siRNA targeting NC (siNC) or YTHDF3 (siYTHDF3‐#1 or ‐#2). N) Immunoprecipitation (with anti‐IgG or anti‐FLAG) and western blotting of SUV39H1, SUV39H2, and FLAG‐CBX1 in SUNE1, HONE1, or HK1 cells expressing FLAG‐CBX1. O) Immunoprecipitation (with anti‐IgG or anti‐H3K9me3) and western blotting of SUV39H1, SUV39H2, and H3K9me3 in SUNE1, HONE1, or HK1 cells. P) Immunoprecipitation (with anti‐H3K9me3) and western blotting of FLAG, *α*‐Tubulin, SUV39H1, SUV39H2, and H3K9me3 in SUNE1 or HK1 cells that overexpressed control vector or FLAG‐CBX1.

To explore the mechanisms underlying the oncogenic role of CBX1 in NPC, we performed RNA‐seq in sgNC and sgCBX1‐1 SUNE1 cells and chromatin immunoprecipitation (ChIP) sequencing (ChIP‐seq) in SUNE1 cells with CBX1 overexpression to identify potential targets of CBX1. The RNA‐seq analysis showed that there were 122 upregulated genes and 50 downregulated genes upon CBX1 knockout (**Figure** **4**A,B). Kyoto Encyclopedia of Genes and Genomes (KEGG) analysis further confirmed that these genes were enriched in cancer‐related signaling pathways (Figure 4C). In addition, a total of 1028 target genes were found to be enriched with CBX1 overexpression through the ChIP‐seq analysis (Figure 4D; Figure S5A, Supporting Information). Combining the RNA‐seq and ChIP‐seq results, we obtained three potential target genes: SLC16A7, ZNF297B and MAP7 (Figure 4E). We next analyzed the expression of these three genes in NPC cells with CBX1 knocked down. CBX1 knockdown significantly promoted MAP7 expression in SUNE1 and HONE1 cells, while there was little change in the expression of SLC16A7 or ZNF297B (Figure S5B‐D, Supporting Information). The downregulation of MAP7 was further confirmed in CBX1‐knockout NPC cells (Figure 4F). Correspondingly, MAP7 protein expression was remarkably enhanced in both CBX1‐knockdown NPC cells and CBX1‐knockout NPC cells (Figure 4G; Figure S5E,F, Supporting Information). The ChIP‐seq analysis showed that there were two CBX1 binding motifs, which could recognize three binding sites in the *MAP7* promoter (Figure 4H; Figure S5G,H, Supporting Information). Further ChIP assays confirmed that CBX1 could directly bind to the promoter of *MAP7* (Figure 4I). These results indicated that MAP7 was the downstream target gene of CBX1 in NPC.

Given that CBX1 is the histone methylation reader that can bind to trimethylated H3K9 (H3K9me3) and contribute to heterochromatin formation,^[^ ^20^ ^]^ we evaluated whether the observed MAP7 downregulation depended on heterochromatin regulation by CBX1 in NPC. We performed ChIP assays and found significant enrichment of H3K9me3 in the MAP7 promoter (Figure 4J). In addition, coimmunoprecipitation (co‐IP) and immunoblot analysis showed an interaction between CBX1 and H3K9me3 (Figure 4K). Moreover, knockout of CBX1 resulted in a decreased level of H3K9me3 and an increased level of MAP7 (Figure 4L; Figure S5F, Supporting Information). Reversely, knocking down YTHDF3 resulted in an increased level of H3K9me3 and a decreased level of MAP7 (Figure 4M). These results suggest that CBX1, regulated by YTHDF3 in an m^6^A‐dependent manner, transcriptionally inhibits the expression of MAP7 through the formation of H3K9me3 in NPC cells. As previous reports^[^ ^21^ ^]^ have suggested that the methyltransferases SUV39H1 and SUV39H2 are the major enzymes involved in the formation of H3K9me3, we then examined whether CBX1 can interact with SUV39H1 or SUV39H2 in NPC cells. The results showed that CBX1 was coimmunoprecipitated with both SUV39H1 and SUV39H2 in NPC cells (Figure 4N). Further co‐IP assays validated that SUV39H1 and SUV39H2 also interacted with H3K9me3 (Figure 4O). In addition, overexpression of CBX1 enhanced the interaction between H3K9me3 and SUV39H1/2 (Figure 4P), indicating that CBX1 recruits SUV39H1/2 to form H3K9me3 on chromatin in NPC cells. Together, these results indicate that CBX1 induces transcriptional inhibition of MAP7 through H3K9me3‐mediated heterochromatin formation in NPC.

### 2.5 Enhanced Binding of CBX1 to H3K9me3 Facilitates MAP7 Inhibition

**Figure 5:**
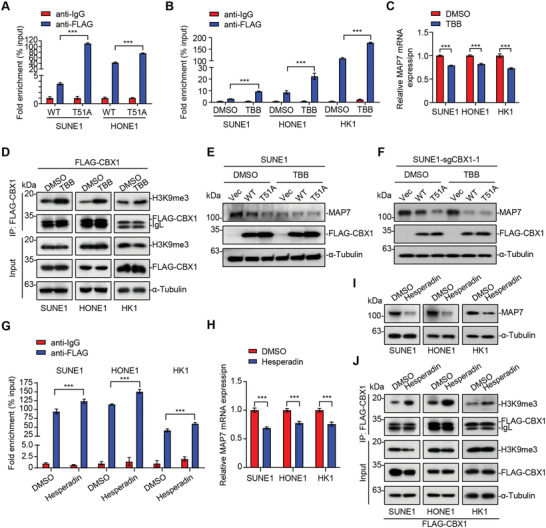
Enhanced binding of CBX1 to H3K9me3 facilitates MAP7 inhibition. A) qPCR analysis of CBX1 occupation on MAP7 binding sites following by ChIP experiment (anti‐IgG or anti‐FLAG) in SUNE1 and HONE1 cells expressing FLAG‐tagged wild type (WT) or mutant (T51A) CBX1. Mean (*n* = 3) ± s.d. Two‐way ANOVA, *** *P* < 0.001. B) qPCR analysis of CBX1 occupation on MAP7 binding sites following by ChIP experiment (anti‐IgG or anti‐FLAG) in SUNE1, HONE1 or HK1 cells expressing FLAG‐CBX1 treated with DMSO or TBB for 6 h (75 × 10^−6^ m). Mean (*n* = 3) ± s.d. Two‐way ANOVA, *** *P* < 0.001. C) Relative mRNA levels of MAP7 in SUNE1, HONE1, or HK1 cells treated with DMSO or TBB for 12 h (75 × 10^−6^ m). Mean (*n* = 3) ± s.d. Student's *t*‐test, *** *P* < 0.001. D) Immunoprecipitation (with anti‐FLAG) and western blotting of H3K9me3, FLAG‐CBX1, and *α*‐Tubulin in SUNE1, HONE1, or HK1 cells expressing FLAG‐CBX1 treated with DMSO or TBB for 6 h (75 × 10^−6^ m). E,F) Western blotting of MAP7, FLAG‐CBX1, and *α*‐Tubulin in SUNE1 E) or SUNE1‐sgCBX1‐1 F) cells expressing empty vector or FLAG‐tagged wild type (WT) or mutant (T51A) CBX1 treated with DMSO or TBB for 12 h (75 × 10^−6^ m). G) qPCR analysis of CBX1 occupation on MAP7 binding sites following by ChIP experiment (anti‐IgG or anti‐FLAG) in SUNE1, HONE1, or HK1 cells expressing FLAG‐CBX1 treated with DMSO or Hesperadin for 1 h (500 × 10^−6^ m). Mean (*n* = 3) ± s.d. Two‐way ANOVA, *** *P* < 0.001. H, I) Relative mRNA levels of MAP7 (H) and western blotting (I) of MAP7, FLAG‐CBX1 and *α*‐Tubulin in SUNE1, HONE1 or HK1 cells treated with DMSO or Hesperadin for 24 h (500 × 10^−9^ m). J) Immunoprecipitation (with anti‐FLAG) and western blotting of H3K9me3, FLAG‐CBX1 and *α*‐Tubulin in SUNE1, HONE1 or HK1 cells expressing FLAG‐CBX1 treated with DMSO or Hesperadin for 1 h (500 × 10^−9^ m).

It has been reported that phosphorylation of CBX1 on amino acid Thr51 (T51) can release CBX1 from chromatin around H3K9me3 sites, and inhibition of casein kinase 2 (CK2) by the inhibitor TBB suppresses T51 phosphorylation.^[^ ^19c^ ^]^ We then constructed the T51A mutant, which cannot be phosphorylated by CK2, and performed ChIP assays with NPC cells. The results showed that the T51A mutant was more enriched than wild‐type (WT) CBX1 in the MAP7 promoter (**Figure** **5**A). Supporting this, inhibition of the phosphorylation of T51 by TBB also increased the enrichment of CBX1 in the MAP7 promoter and further inhibited the mRNA expression of MAP7 in NPC cells (Figure 5B,C). We then performed co‐IP assays and found that the interactions between H3K9me3 and CBX1 were enhanced upon TBB treatment (Figure 5D). In addition, overexpression of WT CBX1 significantly inhibited the MAP7 expression compared to that of control vector, and this inhibition was further potentiated by TBB treatment, while overexpression of the T51A mutant substantially inhibited MAP7 expression compared to that of WT CBX1, and this effect was not changed by TBB treatment in SUNE1 cells (Figure 5E). To further confirmed the results, we reconstituted CBX1‐knockout cells with control vector, WT or T51A CBX1, and found that TBB treatment had little effect on MAP7 expression in CBX1‐knockout cells reconstituted with control vector. Moreover, similar results as those in SUNE1 cells were recapitulated in CBX1‐knockout cells reconstituted with WT or T51A CBX1 upon TBB treatment (Figure 5F). Collectively, these results show that the phosphorylation of CBX1 at the T51 site is critical for its binding to H3K9me3 and inhibition of MAP7 expression.

Since phosphorylation of H3S10 by Aurora B results in the dissociation of CBX1 proteins from H3K9me3,^[^ ^20b^ ^]^ we used the inhibitor hesperadin, which targets Aurora B,^[^ ^22^ ^]^ to determine whether inhibition of the phosphorylation of H3S10 would increase the enrichment of CBX1 in the MAP7 promoter. The results showed that hesperadin treatment increased the enrichment of CBX1 in the MAP7 promoter and further resulted in decreased in the mRNA and protein levels of MAP7 in NPC cells (Figure 5G–I). Co‐IP assays also showed stronger interactions between H3K9me3 and CBX1 upon hesperadin treatment (Figure 5J). Together, these data suggest that enhancing the ability of CBX1 to bind to H3K9me3 facilitates the transcriptional repression of MAP7.

### 2.6 MAP7 Acts Downstream of CBX1 in NPC Tumorigenesis

MAP7 is a member of the microtubule‐associated protein family (microtube‐associated proteins, MAPs), which can regulate organelle localization and protein transport by regulating the activity of molecular motors and participates in the transport of organelles and macromolecular substances along microtubules.^[^ ^23^ ^]^ However, the function and mechanism of MAP7 in tumor progression remain largely unknown.

**Figure 6:**
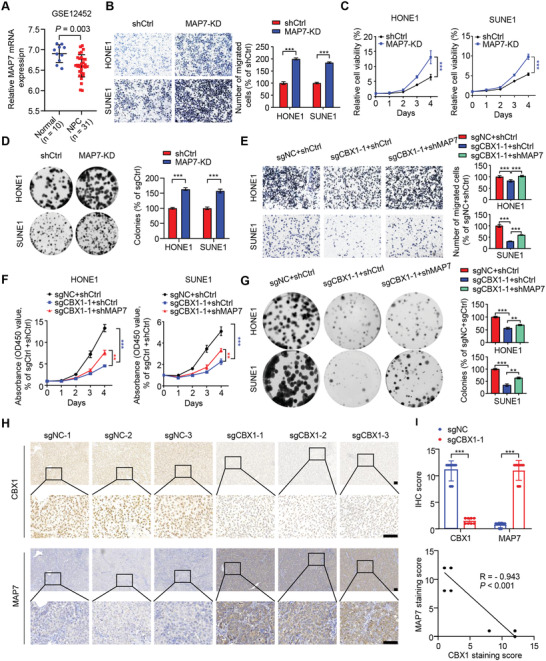
MAP7 acts downstream of CBX1 in NPC tumorigenesis. A) Relative mRNA levels of MAP7 in normal (*n* = 10) and NPC (*n* = 31) tissue samples in the GSE12452 dataset. Student's *t*‐test. B–D) Representative images and quantification of migration transwell assays (B), CCK‐8 assays C) and colony formation assays D) in SUNE1 and HONE1 cells transfected with shRNA targeting control vector (shCtrl) or MAP7 (MAP7‐KD). Mean (*n* = 5 in (B), *n* = 6 in C and *n* = 3 in (D) ± s.d. Student's *t*‐test, *** *P* < 0.001. E–G) Representative images and quantification of migration transwell assays E), CCK‐8 assays F), and colony formation assays G) in SUNE1 and HONE1 cells expressing sgNC or sgCBX1 transfected with shRNA targeting control (shCtrl) or MAP7 (shMAP7). Mean (*n* = 5 in (E), *n* = 6 in (F), and *n* = 3 in (G)) ± s.d. One‐way ANOVA in (E,G), two‐way in (F), *** *P* < 0.001. H,I) Representative images of immunohistochemical staining for CBX1 or MAP7 H) and correlation analysis of CBX1 expression and MAP7 expression according to IHC score statistic I) in the axilla tumors of nude mice from sgNC or sgCBX1 group. Scale bars, 100 µm. *** *P* < 0.001; Student's *t*‐test (up) and Pearson R statistical test (down) were used.

By analyzing the public GSE12452 dataset, we found significantly higher mRNA expression of MAP7 in normal nasopharyngeal epithelial tissues than in NPC tissues (**Figure** **6**A), which was consistent with the above data showing that oncogenic CBX1 transcriptionally repressed the expression of MAP7 in NPC. To investigate the function of MAP7 in NPC, we knocked‐down MAP7 expression in NPC cells by using siRNAs and shRNA (Figure S6A,B, Supporting Information) and performed transwell, CCK‐8 and colony formation assays. The results showed that knocking down MAP7 significantly promoted NPC cell migration, proliferation and colony formation (Figure 6B–D; Figure S6C–E, Supporting Information), which indicated that MAP7 may play an inhibitory role in NPC progression. To further elucidate the function of MAP7, we performed functional studies with CBX1‐knockout cells combined with MAP7 knockdown (Figure S6F,G, Supporting Information). Our data showed that knockdown of MAP7 remarkably rescued the inhibitory effects on malignant phenotypes caused by CBX1‐konckout (Figure 6E–G). These results suggest that MAP7 is a functional target gene of CBX1 in NPC tumorigenesis. Moreover, we evaluated the expression of MAP7 and CBX1 in xenograft tumors and found that MAP7 expression was negatively correlated with CBX1 expression (Figure 6H,I), which further confirmed that CBX1 promoted NPC tumorigenesis by targeting MAP7.

### 2.7 CBX1 Inhibition Decreases IFN‐*γ*‐Inducible PD‐L1 Expression in NPC Cells

**Figure 7:**
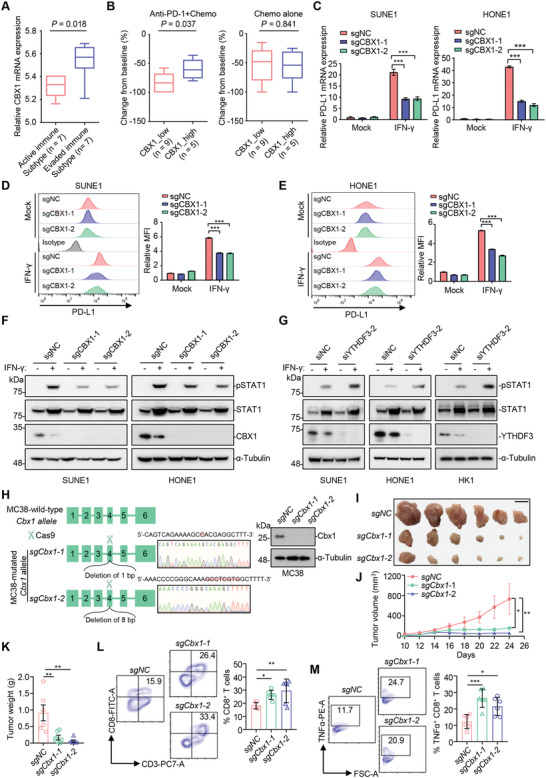
CBX1 inhibition decreases IFN‐*γ*‐inducible PD‐L1 expression in NPC cells. A) Box plots showing relative mRNA levels of CBX1 in active (*n* = 7) and evaded (*n* = 7) immune subtypes of NPC in the EGAS00001004542 dataset. All patients have receiving anti‐PD‐1 plus chemotherapy. The box plot center corresponds to the median, with the box and whiskers corresponding to the interquartile range and 1.5 × interquartile range, respectively. *P*‐values were based on the Wilcoxon rank‐sum test. B) Box plots showing changes from baseline in high versus low *CBX1* expression groups for the 14 NPC patients receiving anti‐PD‐1 plus chemotherapy (left) and the 14 matched patients receiving chemotherapy alone (right) (EGAS00001004542 dataset). The optimal cut‐off value for CBX1 expression was determined by receiver operator characteristic analysis. The box plot center corresponds to the median, with the box and whiskers corresponding to the interquartile range and 1.5 × interquartile range, respectively. *P*‐values were based on the Wilcoxon rank‐sum test. C) Relative mRNA levels of PD‐L1 in SUNE1 and HONE1 cells stably transfected with sgRNA targeting NC (sgNC) or CBX1 (sgCBX1‐1 or ‐2) treated with or without IFN‐*γ* for 24 h (10 ng mL). Mean (*n* = 3) ± s.d. Two‐way ANOVA, *** *P* < 0.001. D,E) Flow cytometry analyzing of PD‐L1 expression in sgNC, sgCBX1‐1, or sgCBX1‐2 SUNE1 or HONE1 cells treated with or without IFN‐*γ* for 24 h (10 ng mL^−1^). MFI, mean fluorescent intensity. Mean (*n* = 3) ± s.d. Two‐way ANOVA, *** *P* < 0.001. F) Western blotting of pSTAT1, STAT1, FLAG‐CBX1, and *α*‐Tubulin in SUNE1 and HONE1 cells stably transfected with sgRNA targeting NC (sgNC) or CBX1 (sgCBX1‐1 or ‐2) treated with or without IFN‐*γ* for 24 hs (10 ng mL^−1^). G) Western blotting of pSTAT1, STAT1, FLAG‐CBX1 and *α*‐Tubulin in SUNE1, HONE1, or HK1 cells transfected with siRNA targeting NC (siNC) or YTHDF3 (siYTHDF3‐1) treated with or without IFN‐*γ* for 24 h (10 ng mL^−1^). H) Strategy and amplicon sequencing of CRISPR/Cas9‐mediated editing of mouse *Cbx1* gene and western blotting of Cbx1 and *α*‐Tubulin in WT (sgNC) or Cbx1‐knockout (*sgCbx1*‐1 or ‐2) MC38 cells. I–M) WT or Cbx1‐knockout MC38 cells were injected into the axilla of C57BL/6J mice to construct tumor growth model. Images I), tumor growth curves J) and weight K) of tumors. The proportion of CD8*^+^* T cells among CD3^+^CD45^+^ T cells L) and TNF*α* ^+^ T cells among CD3^+^CD45^+^ CD8^+^ T cells M) was determined by flow cytometry. Mean (*n* = 6) ± s.e.m. One‐way ANOVA, * *P* < 0.05, ** *P* < 0.01, *** *P* < 0.001.

Given that CBX1 exerts an inhibitory effect on the NPC‐cell killing by CAR‐T cells (Figure 2H,I), we evaluated its role in immunomodulation. We first assessed CBX1 mRNA expression in the active and evaded immune subtypes of NPC, which were classified in our previous study.^[^ ^24^ ^]^ In the 14 NPC patients who received anti‐PD‐1 therapy plus chemotherapy in our EGAS00001004542 dataset,^[^ ^24^ ^]^ we found a significantly higher level of *CBX1* expression in the evaded immune subtype than in the active immune subtype (**Figure** **7**A). Furthermore, we identified significantly better tumor shrinkage within the CBX1‐high group among the patients receiving anti‐PD‐1 therapy plus chemotherapy, while no differences were identified between the CBX1‐high and CBX1‐low groups for the matched patients receiving chemotherapy alone (Figure 7B). These results suggest a potential role for CBX1 in NPC immune evasion. As immunosuppressive molecules (e.g., PD‐L1, IDO1, and IL‐18BP) that can be induced by interferon‐gamma (IFN‐*γ*) are generally important to facilitate immune evasion by tumor cells,^[^ ^25^ ^]^ we wondered whether the expression of these immunosuppressive molecules in NPC cells is affected by CBX1. qPCR results revealed that CBX1 knockout significantly suppressed PD‐L1 mRNA expression upon IFN‐*γ* stimulation, while no significant effects on the mRNA expression of IDO1 or IL‐18BP were found (Figure 7C; Figure S7A,B, Supporting Information). Moreover, knockdown of CBX1 significantly suppressed PD‐L1 mRNA expression upon IFN‐*γ* stimulation (Figure S7C,D, Supporting Information). We further investigated the effect of CBX knockout and knockdown on the surface expression of PD‐L1 upon IFN‐*γ* stimulation using flow cytometry and observed the same phenomenon (Figure 7D,E; Figure S7E, Supporting Information).

It has been reported that IFN‐*γ* can activate the STAT1 signaling pathway, ultimately upregulating the expression of PD‐L1.^[^ ^25c^ ^]^ We found that CBX1 knockout or knockdown significantly suppressed the phosphorylation of STAT1 upon IFN‐*γ* stimulation (Figure 7F; Figure S7F, Supporting Information). Moreover, knocking down YTHDF3 remarkably promoted STAT1 phosphorylation and PD‐L1 expression upon IFN‐*γ* stimulation (Figure 7G; Figure S7G, Supporting Information). Together, these results indicated a negative correlation between CBX1 expression and the anti‐PD‐1 response and suggested that targeting CBX1 could inhibit IFN‐*γ*‐STAT1 signaling‐induced PD‐L1 expression, which might further suppress immune evasion in NPC.

To further investigate the in vivo function of CBX1 on CD8^+^ T cells, we generated Cbx1‐knockout MC38 cells (Figure 7H) and established a MC38‐C57BL/6J mouse model. The results showed that knockout of CBX1 significantly inhibited the growth of MC38 tumor (Figure 7I–K). In addition, the proportion of tumor‐infiltrating CD8^+^ T cells as well as TNF*α* ^+^ CD8^+^ T cells was significantly increased in Cbx1‐knockout tumors (Figure 7L,M), indicating the enhanced CD8^+^ T‐cell antitumor immune response in Cbx1‐knockout tumors.

### 2.8 CBX1 Serves as an Independent Predictor for Unfavorable Prognosis in NPC Patients

**Figure 8:**
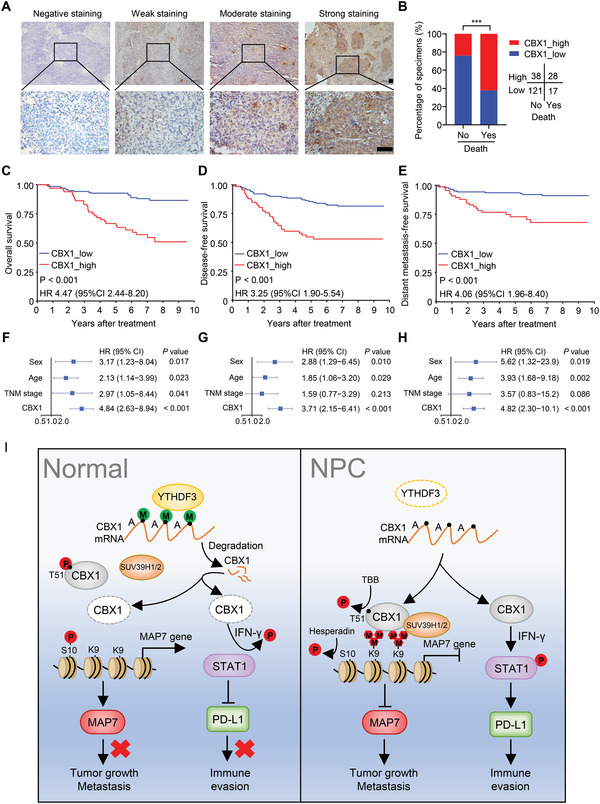
High expression of CBX1 is associated with a poor prognosis in NPC patients. A) Representative image of IHC staining for CBX1 graded according to the intensity of staining in NPC tissue samples. Scale bars, 50 µm. B) Correlations of death status with the level of CBX1 expression detected by IHC. The *P* value was determined using chi‐square (*χ*2) test. C–E) Kaplan–Meier analysis of overall survival C), disease‐free survival D) and distant metastasis‐free survival E) according to the CBX1 expression levels in 204 NPC patients. The *P* values in (C) to (E) were determined using the log‐rank test. F–H) Forest plots of multivariate Cox regression analyses showing the significance of different prognostic variables in NPC overall survival F), disease‐free survival G), and distant metastasis‐free survival H). I) Proposed working model of the m^6^A‐modified CBX1 on NPC progression.

Finally, we conducted immunohistochemical (IHC) staining of 204 NPC tissue samples with an antibody against CBX1 to determine the clinical relevance of CBX1 in NPC patients. We observed CBX1 expression in both the cytoplasm and the nucleus and then divided the samples into four groups according to staining intensity (negative, weak, moderate, and strong) (**Figure** **8**A). IHC scores were further calculated according to the formula “positive rate score × staining intensity score”, and the best cutoff value was chosen by receiver operator characteristic (ROC) curve analysis to divide the NPC patients into high and low CBX1 expression groups. By incorporating clinical data into the analysis, we found that higher CBX1 expression was significantly correlated with higher risks of death, disease and metastasis (Figure 8B; Table S2, Supporting Information). Further Kaplan–Meier analysis revealed that higher CBX1 expression indicated poorer overall survival, disease‐free survival and distant metastasis‐free survival (Figure 8C–E). Multivariate analysis identified CBX1 as an independent prognostic predictor for NPC prognosis (Figure 8F–H). Taken together, our findings demonstrate that higher expression of CBX1 predicts a more unfavorable prognosis in NPC patients.

## 3 Discussion

In the present study, we identified an m^6^A‐regulated histone methylation regulator CBX1 that was significantly upregulated with m^6^A hypomethylation in NPC. m^6^A‐modified CBX1 mRNA was recognized and destabilized by the m^6^A reader YTHDF3. Furthermore, we revealed that CBX1 acted as an oncogene through epigenetic remodeling in NPC, which promoted NPC cell migration, invasion and proliferation through transcriptional repression of MAP7 via H3K9me3‐mediated heterochromatin formation. These results indicated crosstalk between epitranscriptomic and epigenetic regulation in NPC tumorigenesis. In addition to the oncogenic effect, a negative correlation was found between CBX1 expression and the anti‐PD‐1 response, in which CBX1 was found to facilitate immune evasion through PD‐L1 upregulation mediated by IFN‐*γ*‐STAT1 signaling, and targeting CBX1 enhanced the NPC‐cell killing by CAR‐T cells. Clinically, we identified CBX1 as an independent prognostic predictor of unfavorable survival in NPC patients. Our findings shed light on the function of CBX1 in tumorigenesis and immunomodulation and suggest that CBX1 may be an effective therapeutic target in NPC.

It is widely acknowledged that intricate crosstalk among epigenetic modulators is central to precise and synchronized epigenetic regulation.^[^ ^26^ ^]^ As the mediator of the most common epitranscriptomic modification, the m^6^A machinery is similar to a storm center that frequently interacts with other epigenetic regulators, the coordination of which elicits epigenetic remodeling and accounts for the perplexing modulations of physiological or pathological processes.^[^ ^14^ ^]^ Recent studies have linked histone methylation with m^6^A regulators. For example, it has been reported that METTL14 can regulate embryonic neural stem cell self‐renewal through trimethylation of histone H3 at lysine 4 (H3K4me3, associated with gene activation) and at lysine 27 (H3K27me3, associated with gene repression).^[^ ^27^ ^]^ Moreover, histone H3 trimethylation at Lys36 (H3K36me3), a marker for transcription elongation, can guide m^6^A deposition globally in mouse embryonic stem cells.^[^ ^28^ ^]^ To the best of our knowledge, the present study reveals the direct impact of m^6^A on H3K9me3 (a marker for heterochromatin) in tumorigenesis for the first time. Our findings showed that H3K9me3‐mediated heterochromatin formation was facilitated by the histone methylation reader CBX1 in NPC proliferation and metastasis, in which CBX1 was upregulated with m^6^A hypomethylation, and that m^6^A‐modified CBX1 mRNA was recognized and destabilized by YTHDF3 directly. These findings expand our knowledge on the critical role of crosstalk between regulators involved in transcript biosynthesis and processing during tumorigenesis, which provides potential therapeutic targets.

CBX1, belonging to the heterochromatin‐binding protein family, plays essential roles in the establishment and maintenance of chromatin structures and gene silencing,^[^ ^20^, ^29^ ^]^ thus functioning in multiple biological processes, such as DNA repair, gene transcription and telomere maintenance.^[^ ^19^, ^30^ ^]^ In recent years, accumulating evidence has indicated that CBX plays important roles as a tumor suppressor gene or oncogene in tumor progression.^[^ ^31^ ^]^ For example, CBX1 can suppress metastasis of colorectal cancer cells by decreasing the expression and activation of MMP2.^[^ ^32^ ^]^ Besides, in prostate cancer, CBX1 enhances the activity of androgen receptor to promote cell proliferation.^[^ ^33^ ^]^ High expression of CBX1 was also correlated with a poor prognosis in lung cancer^[^ ^34^ ^]^ and liver cancer.^[^ ^35^ ^]^ Our present study shows that CBX1 plays an important role in NPC tumorigenesis by promoting NPC cell proliferation, migration and invasion and serves as an independent unfavorable prognostic predictor for NPC patients. Mechanistically, it has been reported that CBX1 can function as a histone methylation reader to mediate gene repression by binding H3K9me3 and forming heterochromatin.^[^ ^20^, ^36^ ^]^ Consistent with this, we identified MAP7 as a target gene of CBX1 in NPC, in which MAP7 was transcriptionally repressed via heterochromatin formation mediated by the binding of CBX1 to H3K9me3. Inhibiting the phosphorylation of CBX1 at T51 or the phosphorylation of H3S10 could enhance the binding of CBX1 to H3K9me3 and further facilitate MAP7 inhibition. The regulation of MAP7 expression by miR‐16 was observed in previous studies,^[^ ^37^ ^]^ but this is the first study, to the best of our knowledge, to investigate MAP7 repression by histone methylation. MAP7 mainly functions in protein transport and organelle localization.^[^ ^23^, ^38^ ^]^ Recently, MAP7 was also reported to be involved in tumor progression. In lung cancer, high expression of MAP7 was correlated with favorable overall survival.^[^ ^39^ ^]^ In contrast, MAP7 was overexpressed and positively correlated with a poor prognosis in cervical cancer^[^ ^40^ ^]^ and acute myeloid leukemia.^[^ ^41^ ^]^ Here, we showed that knockdown of MAP7 in NPC promoted cell proliferation, migration and invasion and rescued the inhibition of malignant phenotypes in CBX1‐depleted cells. Moreover, the expression of MAP7 was negatively correlated with CBX1 expression in tumor tissues from a subcutaneous xenograft mouse model. These results suggest a tumor‐suppressive role for MAP7 in NPC. Abrogating the interaction of CBX1 with H3K9me3 to restore MAP7 expression may be a relevant therapeutic intervention for the treatment of NPC.

In addition to the oncogenic effect, the immunomodulatory function of CBX1 was identified in this study. Previous reports have shown that CBXs may be clinically correlated with immune infiltration,^[^ ^42^ ^]^ but the exact role of CBX1 in immunomodulation remains unknown. Our study uncovers that CBX1 promotes immune evasion by NPC cells, which is the first evidence that CBX1 is involved in immunomodulation. We further revealed that IFN‐*γ*‐STAT1 signaling was regulated by CBX1 to facilitate PD‐L1 expression. PD‐L1 expression plays an essential role in immune evasion; thus, its regulation represents an important research topic.^[^ ^43^ ^]^ IFN‐*γ*‐STAT1 signaling has been determined to be the main pathway that initiates PD‐L1 expression in both immune cells and tumor cells, which is well monitored by various molecules, including TET1/2 and A20.^[^ ^25^, ^44^ ^]^ In canonical IFN‐*γ*‐STAT1 signaling, STAT1 is phosphorylated and activated upon the engagement of the receptor IFNGR by IFN‐*γ*, and then activated STAT1 translocates into the nucleus and further binds to DNA to initiate transcription of its target genes, such as PD‐L1.^[^ ^45^ ^]^ Our findings demonstrated that CBX1 inhibition impaired I IFN‐*γ*‐induced STAT1 activation and PD‐L1 expression and thus enhanced the NPC‐cell killing by CAR‐T cells. Therefore, targeting CBX1 simultaneously inhibits tumorigenesis and disrupts the immunosuppressive phenotype in NPC, which may synergize with PD‐L1/PD‐1 blockade, considering the unsatisfactory response rate for immunotherapy in NPC.^[^ ^3^ ^]^ Nevertheless, we acknowledge the limitation that in the present study we examined the immunomodulatory effects of CBX1 in vivo using the MC38 immune model due to the lack of murine NPC cells. Besides, the underlying mechanism by which CBX1 induces PD‐L1 expression needs to be further elucidated.

In summary (Figure 8I), we identified the m^6^A‐regulated histone methylation reader CBX1 and found that m^6^A‐modified CBX1 mRNA could be recognized and destabilized by the m^6^A reader YTHDF3. CBX1 promoted the proliferation, migration, and invasion of NPC cells by transcriptionally repressing MAP7 expression via H3K9me3‐mediated heterochromatin formation, highlighting the integration of epitranscriptomic and epigenetic signaling to tune gene expression. In addition to its oncogenic effect, CBX1 showed an immunomodulatory effect that facilitated immune evasion through PD‐L1 upregulation mediated by IFN‐*γ*‐STAT1 signaling. We also identified the clinical value of CBX1 in representing as an independent predictor for an unfavorable prognosis of NPC patients. These results shed light on the molecular mechanism underlying the effects of CBX1 on NPC progression and may provide a foundation for developing predictive biomarkers and therapeutic targets in NPC.

## 4 Experimental Section

### Clinical Specimens

Four pairs of fresh‐frozen NPC tissue samples with or without metastasis were obtained for m^6^A‐seq and RNA‐seq; the tissues were matched by sex, age, T stage, N stage, and treatment to avoid the influence of these parameters on metastasis. In addition, 204 paraffin‐embedded NPC tissue samples with long‐term follow‐up data and 23 paraffin‐embedded NPC tissue samples without follow‐up data collected between 2004 and 2014 were obtained from the Sun Yat‐sen University Cancer Center (Guangzhou, China) and used for clinical validation. None of the patients who provided tissue samples had been treated with anticancer therapies before biopsy. The clinical features of selected patients are shown in Table S2 (Supporting Information). Informed consent was exempted in this study, which was approved by the Institutional Ethical Review Boards of the Sun Yat‐sen University Cancer Center (G2021‐037‐01).

### Cell Culture

SUNE1, HONE1, CNE1, CNE2, HK1, and HNE1 NPC cells were cultured in RPMI‐1640 medium containing 10% fetal bovine serum (FBS; ExCell Bio, China), and NP69 normal nasopharyngeal epithelial cells were cultured in keratinocyte serum‐free medium supplemented with bovine pituitary extract. The NP69 cell line and all the NPC cell lines were obtained from Professor Musheng Zeng (Sun Yat‐sen University Cancer Center). HEK293T cells were cultured in DMEM medium containing 10% FBS and obtained from American Type Culture Collection (ATCC). All the cells were tested for mycoplasma contamination and cultured for no more than two months.

### In Vivo Animal Experiments

Female BALB/c nude mice and C57BL/6J (4–6 weeks old) were purchased from Beijing Vital River Experimental Animal Technology (Beijing, China) and used in experiments. Establishment and analysis of the inguinal lymph node and lung metastasis models and subcutaneous xenograft model were performed as previously described.^[^ ^15^, ^46^ ^]^ For the inguinal lymph node metastasis model, 3 × 10^5^ SUNE1 cells were injected into the footpads of mice. After 35 days, the mice were sacrificed, and the primary footpad tumors and draining inguinal lymph nodes were collected. For the lung metastasis model, 1 × 10^6^ SUNE1 cells were injected into the tail vein of mice. After 60 days, the mice were sacrificed, and the lungs were collected. For the subcutaneous xenograft model, 1 × 10^6^ SUNE1 cells were injected into the axillary epidermis of mice, and tumor size was monitored every day. After 30 days, the mice were sacrificed, and the tumors were collected and weighed. All the tumors were fixed, embedded in paraffin, and sectioned for further analysis. For the MC38 subcutaneous xenograft model, 1.5 × 10^6^ MC38 cells were injected into the axillary epidermis of C57BL/6J mice, and tumor size was monitored every day. After 25 days, the mice were sacrificed, and the tumors were collected, weighed and prepared into single cell suspensions for flow cytometry. All the animal experiments were approved by the Institutional Animal Care and Use Committee (Sun Yat‐sen University Cancer Center, L102012021000Z).

### Constructs, Antibodies, and Reagents

CBX1 and mutant CBX1 (T51A) were cloned into a Phage‐puro‐6tag vector via standard molecular methods. The Phage‐puro‐6tag vector was obtained as previously described.^[^ ^47^ ^]^ Site‐directed mutagenesis was performed with bridge‐PCR, and sequencing was performed for confirmation. The PSPAX2 (12260) and PMD2.G (12259) plasmids were purchased from Addgene (USA). shRNAs specific for MAP7, YTHDF1 and YTHDF2 were cloned into a pLKO.1 vector (TranSheepBio, Shanghai, China). CBX1 sgRNAs were cloned into a pLenti‐CRISPRv2 vector, which was a kind gift from Dr. Hua Zhang (Sun Yat‐sen University Cancer Center). YTHDF3‐, MAP7‐specific siRNAs were purchased from Ribo Biotechnology (Guangzhou, China). The shRNA, sgRNA and siRNA sequences are listed in Table S3 (Supporting Information).

Anti‐FLAG (F1804, Sigma), horseradish peroxide (HRP)‐conjugated goat anti‐mouse IgG (7076, Cell Signaling Technology, Boston, MA, USA), HRP‐conjugated goat anti‐rabbit IgG (7074, Cell Signaling Technology), anti‐*α*‐tubulin (11224‐1‐AP, Proteintech, Wuhan, Hubei, China), anti‐CBX1 (10241‐2‐AP, Proteintech), anti‐MAP7 (13446‐1‐AP, Proteintech), anti‐YTHDF3 (sc‐377119, Santa Cruz, USA), anti‐SUV39H1 (10574‐1‐AP, Proteintech), anti‐SUV39H2 (11338‐1‐AP, Proteintech), a Pierce™ Magnetic ChIP Kit (26157, Thermo, Waltham, MA, USA), puromycin (A1113802, Thermo), human IFN‐gamma (IFN‐*γ*, 300‐02‐20, PeproTech), APC anti‐human CD274 (329707, Biolegend), PE/Cyanine7 antimouse CD3 (100320, Biolegend), Brilliant Violet 650 anti‐mouse CD45 (103151, Biolegend), FITC anti‐mouse CD8*α* (100706, Biolegend), PE antimouse TNF*α* (506306, Biolegend), anti‐H3K9me3 (ab8898, Abcam), an Annexin V‐FITC/propidium iodide (PI) staining kit (KGA108, KeyGEN, Beijing, China), a Magna RIP kit (17‐700, Millipore, Billerica, MA, USA), a Magna MeRIP m^6^A Kit (17‐10499, Millipore), a RosetteSep Human CD8^+^ T Cells kit (15023, STEMCELL, USA), Dynabeads Human T‐Activator CD3/CD28 (11131D, Invitrogen, CA, USA), hesperadin (HY‐12054, MCE, NJ, USA), TBB (S5265, Sellect, Shanghai, China), Act‐D (Sigma‐Aldrich, USA) were purchased from the indicated manufactures.

### m^6^A‐seq

m^6^A‐seq was conducted by LC‐Bio Technologies (Hangzhou). Briefly, total RNA was extracted from 4 paired metastatic and nonmetastatic NPC tissues using TRIzol reagent. Approximately 25 µg of total RNA was used to deplete ribosomal RNA and was then fragmented into ≈100‐nt oligonucleotides. Then, the cleaved RNA fragments were incubated with an anti‐m^6^A antibody (202003, Synaptic Systems, Germany) in IP buffer supplemented with BSA. The mixture was then incubated with protein‐A beads, eluted and precipitated with ethanol. The eluted m^6^A‐containing fragments (IP) and untreated input control fragments were converted to a final cDNA library in accordance with strand‐specific library preparation by the dUTP method. The average insert size for the paired‐end libraries was ≈100 bp. Then, paired‐end 2 × 150 bp sequencing was performed on an Illumina Novaseq 6000 platform at LC‐BIO Biotech Ltd. (Hangzhou, China) following the vendor's recommended protocol.

### RNA Isolation, Quantitative RT‐PCR and RNA‐seq

RNA extraction was performed by using a RNeasy kit (R0027, Beyotime). First‐strand cDNA was generated by using a reverse transcription kit (Promega). A qRT‐PCR assay was run on a Bio‐Rad SFX (96 or 384) system with 2 × SYBR Green mix (Life, Carlsbad, CA, USA). The data were normalized to the expression of GAPDH. The sequences of the primers are listed in Table S3 (Supporting Information). For RNA‐seq, total RNA was extracted from 3 independent sgNC and sgCBX1‐2 SUNE1 cell samples using TRIzol reagent.

### m^6^A‐RIP Assay

A Magna MeRIP m^6^A Kit (17‐10499, Millipore) was used to perform the m^6^A‐RIP assay according to the manufacturer's instructions. Briefly, NP69, SUNE1, HONE1 and HK1 cells were collected, and ≈300 µg of total RNA was extracted from each cell line. The total RNA was shredded to ≈200 nt and then precipitated and purified by using glycogen/ethanol. Ten percent of the purified RNA was removed as the input, and the remaining RNA was incubated with an anti‐m^6^A antibody or IgG together with magnetic beads. The magnetic bead‐bound complexes were washed, and the RNA was extracted and eluted for purification with an RNA purification kit. Equal volumes of purified RNA were used for reverse transcription PCR, and cDNA was obtained for further qRT‐PCR analysis. Three pairs of primers were designed according to the m^6^A‐seq analysis. The sequences of the primers are listed in Table S3 (Supporting Information). Results were calculated according to the formulas Δ Ct = Ct_IP_ – Ct_Input_, ΔΔ Ct = Δ Ct_m6A_− Δ CT_IgG_ and fold enrichment = 2 (^− Δ Δ Ct^).

### RIP Assay

A Magna RIP RNA‐Binding Protein Immunoprecipitation Kit (17‐700, Millipore) was used to perform the RIP assay according to the manufacturer's protocol. Briefly, cells were lysed in RIP lysis buffer. The lysates were collected, immunoprecipitated with an anti‐YTHDF3 antibody and incubated with protein A/G magnetic beads. The magnetic bead‐bound complexes were immobilized with a magnet, and unbound materials were washed away. The bound RNA was extracted for analysis by qRT‐PCR. The primers used are listed in Table S3 (Supporting Information).

### ChIP Assay

The ChIP assay was performed as previously described.^[^ ^15b^ ^]^ A Pierce Magnetic ChIP Kit (26157, Life) was used to perform the ChIP assay according to the manufacturer's protocol. Briefly, cells were fixed, quenched and lysed. The lysates were digested, and the chromatin was sonicated to produce fragments with a length of ≈500 nt, which were incubated with normal rabbit IgG, anti‐FLAG‐CBX1, anti‐H3K9me3, or anti‐RNA polymerase II and then incubated with ChIP‐grade protein G magnetic beads. The DNA was eluted and purified using a DNA Clean‐UP column and DNA column wash buffer. The purified DNA was used to perform qRT‐PCR (ChIP‐qRT‐PCR) detection or sequencing (ChIP‐seq). The sequences for the ChIP‐qRT‐PCR primers are listed in Table S3 (Supporting Information).

### RNA Pull‐Down

The WT and mutants (A‐T, A‐G, and A‐Delta) of CBX1 fragments were synthesized and constructed into PCDNA 3.1+ plasmids by Tsingke biotechnology (Beijing, China), which were in vitro transcribed into RNA by using the MEGAscript T7 Transcription Kit (AM1334, Thermo, Waltham, MA, USA), and these RNAs were further biotin‐labeled by using the Pierce RNA 3′‐End Desthiobiotinylation Kit (20163, Thermo Fisher Scientific). Cell lysates were extracted from HK1 and SUNE1 cells, and further incubated with the biotin‐labeled RNAs according to the instructions of the Magnetic RNA‐Protein Pull‐Down Kit (20 164, Thermo). The bound proteins were detected by western blotting assay.

### GSEA

GSEA was conducted using the gene expression profiles of 113 NPC samples (GSE102349) to identify differential gene sets between high and low CBX1 mRNA expression groups stratified by the median value. C2 (curated gene sets) and C7 (immunologic signature gene sets) obtained from the Molecular Signatures Database v7.2 were used for enrichment analysis.

### CRISPR/Cas9‐Mediated CBX1 Knockout

The PMD2.G and PSPAX2 packaging plasmids and lentiCRISPRv2‐sgNC or sgCBX1 were cotransfected into HEK293T cells. Forty‐eight hours after transfection, the medium containing the lentivirus was collected. After being filtered through a 0.45 µm filter, the supernatant was transferred to a 50 mL concentration column and centrifuged at 5000 × *g* and 4 °C for 30 min. The concentrated lentivirus was collected and stored at −80 °C. SUNE1, HONE1, or MC38 cells were seeded in a 24‐well plate. Twelve hours later, 20 µL of concentrated lentivirus was added to each well. Forty‐eight hours after infection, the cells were subcultured in selection medium containing 1 µg mL^−1^ puromycin. After 1 week of selection, the cells were seeded in 96‐well plates for further monoclonal cell line constitution. All edited cell lines were validated for knockout efficiency by western blot and amplicon sequencing of targeted loci. For amplicon sequencing‐based validation of knockout efficiency, PCR primers were designed surrounding target sites for human *CBX1* and mouse *Cbx1* sgRNAs. Genomic DNA was isolated from edited cells, and targeted loci were PCR‐amplified, cloned into pUCm‐T vector (Sangon Biotech) and analyzed by sequencing. The sgRNAs used are listed in Table S3 (Supporting Information).

### Cell Migration, Invasion, Colony Formation, and Viability Assays

Cell migration, invasion and colony formation assays were performed as previously described.^[^ ^15^, ^48^ ^]^ For the cell viability assay, SUNE1 (1000), HK1 (2000), or HONE1 (400) cells were seeded into a 96‐well plate (NEST biotechnology) with full medium supplemented with 10% FBS, and cultured for 0–4 days. and stained with a CCK‐8 kit (Dojindo, Tokyo, Japan). The absorbance per well was read on a spectrophotometer at 450 nm.

### CAR‐T Cell Cytotoxicity Assay

CD8^+^ T cells were isolated from human peripheral blood using a RosetteSep human CD8^+^ T‐cell isolation kit. The cells were then cultured and activated by incubation with anti‐CD3/CD28 antibodies for 24 h. The activated CD8^+^ T cells were seeded in RetroNectin‐pretreated plates, and then a HER2‐CAR lentivirus was added to the plates. The medium was replaced after 6 h, and the cells were cultured for another 72 h. The constructed HER2‐CAR‐expressing CD8^+^ T cells were evaluated by flow cytometry. sgNC and sgCBX1 SUNE1 or HONE1 cells were seeded in 6‐well plates, and after the cells had adhered to the plates, CAR‐T cells were added at a 1:1 or 2:1 effector‐to‐target cell ratio and incubated for 24 h at 37 °C. Whole cells were collected for Annexin‐V/PI and anti‐CD3 staining and further flow cytometric analysis.

### Western Blot Analysis

Cells were harvested and lysed in cell lysis buffer (P0013, Beyotime). The following procedures were described previously.^[^ ^48^ ^]^ Briefly, the lysates were centrifuged, and then the supernatants were mixed with SDS loading buffer and heated at 95 °C for 10 min. Afterward, the mixture was subjected to SDS‐PAGE. The proteins were transferred to polyvinylidene fluoride (PVDF) membranes (Millipore), which were subsequently blocked with 5% w/v skim milk. Primary antibodies and secondary antibodies were diluted in the appropriate antibody diluent (Beyotime), and the membranes were incubated with the appropriate antibodies prior to immunoblot analysis.

### Co‐IP Assay

Cells were harvested and lysed in cell lysis buffer (Beyotime), and the lysates were incubated with an anti‐FLAG‐CBX1 antibody overnight at 4 °C. The immune complexes were recovered by incubation with Pierce Protein A/G Magnetic Beads (88802, Thermo Scientific) for 1 h at room temperature and washed three times with wash buffer, followed by western blot analysis.

### IHC Staining and Scoring

Paraffin NPC tissue sections and xenograft mouse tissue sections were evaluated with IHC assays. IHC staining and scoring were performed as previously described.^[^ ^15b^ ^]^ Briefly, tissues were deparaffinized, rehydrated, blocked and subjected to antigen retrieval. Subsequently, nonspecific protein binding was blocked with BSA, and the tissues were incubated with the indicated primary antibodies at 4 °C overnight, labeled with HRP‐conjugated rabbit/mouse secondary antibodies (Dako REALTM EnVision), stained with diaminobenzidine (Sigma) and counterstained with hematoxylin. Images were obtained with an AxioVision Rel.4.6 computerized image analysis system (Carl Zeiss). The staining intensity score was defined as follows: 0, no staining; 1, weak, light‐yellow staining; 2, moderate, yellow‐brown staining; and 3, strong, brown staining. The positive rate score was defined as follows: 1, <10%; 2, 10–35%; 3, 35–70%; and 4, >70%. The total score for the indicated proteins was calculated as the staining intensity score × the positive rate score.

### Survival Analysis

Kaplan–Meier curves were generated using the gene expression profiles of 113 NPC samples (GSE102349) to confirm the prognostic value of CBX1 and YTHDF3 between high and low CBX1 or YTHDF3 mRNA expression groups. The optimal cutoff values for CBX1 and YTHDF3 expression were determined by maximizing Youden's index for PFS by ROC curve analysis. For IHC staining used for CBX1‐related survival analysis, tissues were grouped as either low CBX1 expression (score 0–6) or high CBX1 expression (score 8–12) by maximizing Youden's index for overall survival by ROC curve analysis. Survival curves were plotted by the Kaplan–Meier method and analyzed via the log‐rank test. Multivariate analysis with a Cox proportional hazards model was used to test the independent significance of prognostic factors.

### Statistical Analysis

Data from at least three independent assays are presented as the mean ± SD or mean ± s.e.m. Differences between groups were evaluated by an unpaired two‐tailed Student's *t* test, one‐way ANOVA, two‐way ANOVA or Wilcoxon rank‐sum test (*P < 0.05; **P < 0.01; ***P < 0.001; ns, no significance). Pearson *R* statistical test was used in correlation analysis. ROC curve analysis was used to determine optimal cutoff value. The Kaplan–Meier method was used to construct survival curves, and the log‐rank test was used to compare differences among groups. Multivariate analysis using a Cox proportional hazards regression model was applied to identify independent prognostic factors. All statistical analyses were performed using GraphPad Prism 8 or SPSS 20 software. The m^6^A‐seq, RNA‐seq and ChIP‐seq profiles are accessible at the GEO repository under accession number GSE200794. The key raw data were uploaded to the Research Data Deposit public platform (http://www.researchdata.org.cn; RDDB2022470878).

## Conflict of Interest

The authors declare no conflict of interest.

## Authors Contribution

Y.Z and S.Y.H contributed equally to this work. Y.Z. conceived the study, designed, and performed most of the experiments and wrote the manuscript. S.Y.H. constructed the plasmids and performed RNA‐seq and analyzed the data. X.R.T. helped with the analysis of CBX1 prognosis. L.F.L. performed the ChIP and RIP experiments. Q.M.H. performed the IHC staining against CBX1 in NPC patients. X.Y.L. constructed the CAR‐T cells. J.W.B helped with the animal experiments. J.Y.L., Y.Q.L., and Q.J.L performed GSEA and analyzed data. N.L. and J.M. provided regents and discussed the project. Y.P.C. conceived the study and wrote the manuscripts. All authors discussed the data and read the manuscript.

## Supporting information

Supporting Information

## Data Availability

The key raw data were uploaded to the Research Data Deposit public platform (http://www.researchdata.org.cn, RDDB2022470878) and are available from the corresponding author upon reasonable request.
