# Supplementary material for: N6‐Methyladenosine‐Modified CBX1 Regulates Nasopharyngeal Carcinoma Progression Through Heterochromatin Formation and STAT1 Activation
Source: Adv Sci (Weinh). 2022 Oct 30;9(36):2205091. doi: 10.1002/advs.202205091 (PMC9798977; doi:10.1002/advs.202205091)
Supplement: Supplementary file 1 — Supporting Information [file ADVS-9-2205091-s001.pdf]

ADVANCED SCIENCE
Open Access

Supporting Information

for *Adv. Sci.*, DOI 10.1002/advs.202205091

N^6-Methyladenosine-Modified CBX1 Regulates Nasopharyngeal Carcinoma Progression Through Heterochromatin Formation and STAT1 Activation

Yin Zhao, Shengyan Huang, Xirong Tan, Liufen Long, Qingmei He, Xiaoyu Liang, Jiewen Bai, Qingjie Li, Jiayi Lin, Yingqin Li, Na Liu, Jun Ma and Yupei Chen**

Supporting Information

N^6-methyladenosine-modified CBX1 regulates nasopharyngeal carcinoma progression through heterochromatin formation and STAT1 activation

Yin Zhao, Shengyan Huang, Xirong Tan, Liufen Long, Qingmei He, Xiaoyu Liang, Jiewen Bai, Qingjie Li, Jiayi Lin, Yingqin Li, Na Liu, Jun Ma, Yupei Chen**

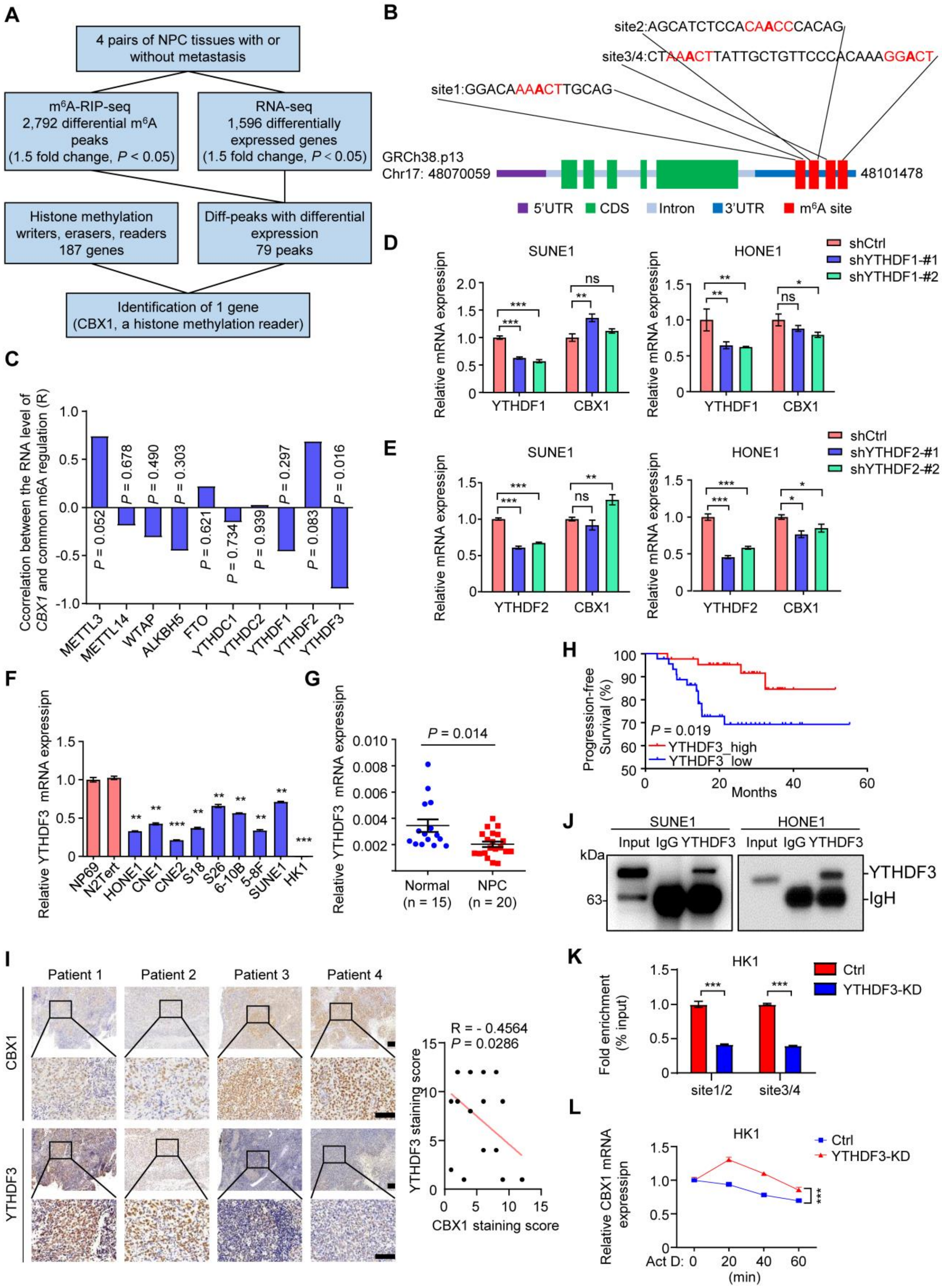
A
4 pairs of NPC tissues with or without metastasis
m6A-RIP-seq
2,792 differential m6A peaks
(1.5 fold change, P < 0.05)
RNA-seq
1,596 differentially expressed genes
(1.5 fold change, P < 0.05)
Histone methylation writers, erasers, readers
187 genes
Diff-peaks with differential expression
79 peaks
Identification of 1 gene
(CBX1, a histone methylation reader)
B
site2:AGCATCTCCACAACCCACAG
site3/4:CTAAACTTATTGCTGTTCCCACAAAGGACT
site1:GGACAAAACTTGCAG
GRCh38.p13
Chr17: 48070059
48101478
5'UTR
CDS
Intron
3'UTR
m6A site
C
Ccorrelation between the RNA level of CBX1 and common m6A regulation (R)
P = 0.052
P = 0.678
P = 0.490
P = 0.303
P = 0.621
P = 0.734
P = 0.939
P = 0.297
P = 0.083
P = 0.016
METTL3
METTL14
WTAP
ALKBH5
FTO
YTHDC1
YTHDC2
YTHDF1
YTHDF2
YTHDF3
D
SUNE1
HONE1
shCtrl
shYTHDF1-#1
shYTHDF1-#2
Relative mRNA expressipn
YTHDF1
CBX1
E
SUNE1
HONE1
shCtrl
shYTHDF2-#1
shYTHDF2-#2
Relative mRNA expressipn
YTHDF2
CBX1
F
Relative YTHDF3 mRNA expressipn
NP69
N2Tert
HONE1
CNE1
CNE2
S18
S26
6-10B
5-8F
SUNE1
HK1
G
Relative YTHDF3 mRNA expressipn
P = 0.014
Normal
(n = 15)
NPC
(n = 20)
H
Progression-free Survival (%)
P = 0.019
YTHDF3_high
YTHDF3_low
Months
I
Patient 1
Patient 2
Patient 3
Patient 4
CBX1
YTHDF3
R = - 0.4564
P = 0.0286
YTHDF3 staining score
CBX1 staining score
J
SUNE1
HONE1
Input
IgG
YTHDF3
kDa
63
YTHDF3
IgH
K
HK1
Ctrl
YTHDF3-KD
Fold enrichment (% input)
site1/2
site3/4
L
HK1
Ctrl
YTHDF3-KD
Relative CBX1 mRNA expressipn
Act D:
(min)

Figure S1. m^6A-modified CBX1 mRNA was recognized and destabilized by the m^6A reader YTHDF3. A) Flow chart for the identification of m^6A modified histone methylation regulator in NPC. B) Schematic representation of positions of m^6A motifs within CBX1 mRNA. C) Correlation between the mRNA expression level of CBX1 and common m^6A regulators in NPC malignant cells (GSE150430 dataset). Seven NPC samples containing a total of 7,126 malignant cells (≥ 200 cells per sample) profiled by single-cell RNA sequencing were analyzed. Pearson R statistical test was used. D) Relative mRNA levels of CBX1 and YTHDF1 in SUNE1 and HONE1 cells transfected with shRNA targeting Control (shCtrl) or YTHDF1 (shYTHDF1 #1 or #2). Mean (n = 3) ± s.d. One-way ANOVA, ns, no significant, * $P < 0.05$, ** $P < 0.01$, *** $P < 0.001$, ns, no significant. E) Relative mRNA levels of CBX1 and YTHDF2 in SUNE1 and HONE1 cells transfected with shRNA targeting Control (shCtrl) or YTHDF2 (shYTHDF2 #1 or #2). Mean (n = 3) ± s.d. One-way ANOVA, ns, no significant, * $P < 0.05$, ** $P < 0.01$, *** $P < 0.001$, ns, no significant. F) Relative mRNA levels of YTHDF3 in NP69, N2Tert, HONE1, CNE1, CNE2, S18, S26, 6-10B, 5-8F, SUNE1 and HK1 cells. Mean (n = 2) ± s.d. One-way ANOVA, ** $P < 0.01$, *** $P < 0.001$. G) Relative mRNA levels of YTHDF3 in normal (n = 15) and NPC (n = 20) patients. Mean (n = 2) ± s.d. Student's *t*-test. H) Kaplan-Meier analysis of progression-free survival according to the YTHDF3 expression levels in the GSE102349 dataset. The *P* values were determined using the log-rank test. I) Representative images of IHC staining for CBX1 and YTHDF3 (n = 23), and correlation analysis of the expression of CBX1 and YTHDF3 according to IHC score statistic. Scale bars, 50 µm. Pearson R statistical test was used. J) RIP (with anti-IgG or anti-YTHDF3) and western blotting of YTHDF3 in SUNE1 and HONE1 cells. K) RIP-qPCR analysis of YTHDF3 occupation in CBX1 m^6A sites in HK1 cells transfected with siRNA targeting NC (Ctrl) or YTHDF3 (YTHDF3-KD). Mean (n = 3) ± s.d. Student's t-test, *** $P < 0.001$. L) Relative mRNA levels of CBX1 in HK1 cells transfected with siRNA targeting NC (siNC) or YTHDF3 (siYTHDF3-KD) followed by treatment with actinomycin D (1 µg/ml) by indicated time. Mean (n = 3) ± s.d. Two-way ANOVA, *** $P < 0.01$.

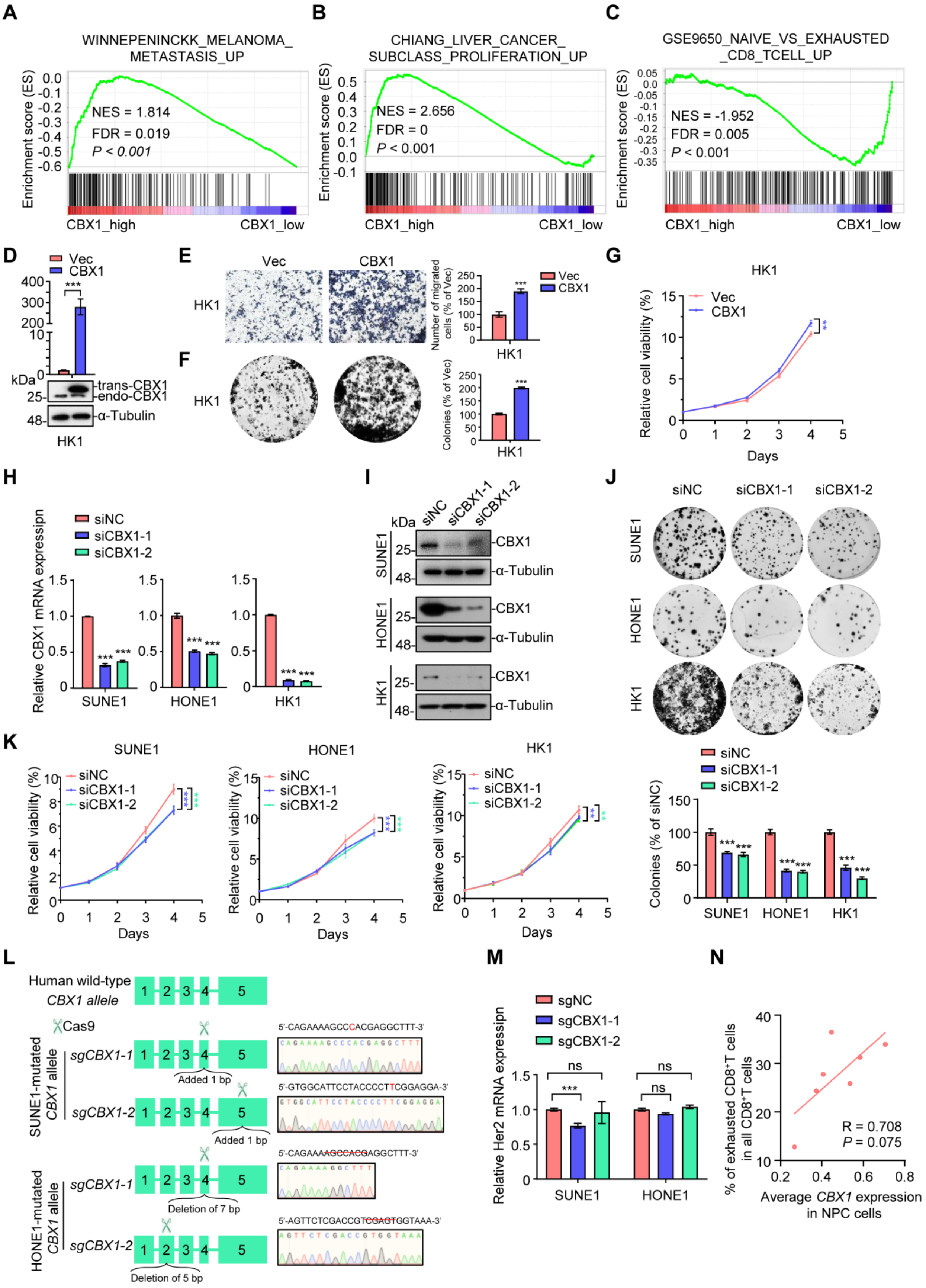
A
WINNEPENINCKK_MELANOMA_METASTASIS_UP
Enrichment score (ES)
NES = 1.814
FDR = 0.019
P < 0.001
CBX1_high
CBX1_low
B
CHIANG_LIVER_CANCER_SUBCLASS_PROLIFERATION_UP
Enrichment score (ES)
NES = 2.656
FDR = 0
P < 0.001
CBX1_high
CBX1_low
C
GSE9650_NAIVE_VS_EXHAUSTED_CD8_TCELL_UP
Enrichment score (ES)
NES = -1.952
FDR = 0.005
P < 0.001
CBX1_high
CBX1_low
D
Vec
CBX1
kDa
trans-CBX1
endo-CBX1
α-Tubulin
HK1
E
Vec
CBX1
HK1
Number of migrated cells (% of Vec)
F
HK1
Colonies (% of Vec)
G
HK1
Relative cell viability (%)
Days
H
siNC
siCBX1-1
siCBX1-2
Relative CBX1 mRNA expressipn
SUNE1
HONE1
HK1
I
kDa
siNC
siCBX1-1
siCBX1-2
SUNE1
HONE1
HK1
CBX1
α-Tubulin
J
siNC
siCBX1-1
siCBX1-2
SUNE1
HONE1
HK1
Colonies (% of siNC)
K
SUNE1
HONE1
HK1
Relative cell viability (%)
Days
L
Human wild-type CBX1 allele
Cas9
SUNE1-mutated CBX1 allele
sgCBX1-1
sgCBX1-2
Added 1 bp
HONE1-mutated CBX1 allele
Deletion of 7 bp
Deletion of 5 bp
5'-CAGAAAAGCCCACGAGGCTTT-3'
5'-GTGGCATTCCTACCCCTTCGGAGGA-3'
5'-CAGAAAAGCCCACGAGGCTTT-3'
5'-AGTTCTCGACCGTCGAGTGGTAAA-3'
M
sgNC
sgCBX1-1
sgCBX1-2
Relative Her2 mRNA expressipn
ns
SUNE1
HONE1
N
% of exhausted CD8⁺T cells in all CD8⁺T cells
R = 0.708
P = 0.075
Average CBX1 expression in NPC cells

Figure S2. Knockdown of CBX1 suppresses NPC cell proliferation. A-C) GSEA analysis based on GSE102349 dataset showing metastasis- (A), proliferation- (B) and CD8 T cell- (C) related biological functions enriched in response to high CBX1 expression. D) Relative mRNA level of CBX1 and protein levels of CBX1 and α-Tubulin in HK1 cells that overexpressed control vector or FLAG-CBX1. Mean (n = 3) ± s.d. Student's *t*-test, *** $P < 0.001$. E-G) Representative images and quantification of transwell assay (E, without Matrigel), colony formation assays (F) and CCK-8 assays (G) in HK1 cells overexpressed control vector or FLAG-CBX1. Mean (n = 5 in E, n = 3 in F and n = 6 in G) ± s.d. Student's *t*-test in (D, E, F), Two-way ANOVA in (G); *** $P < 0.001$. H, I) Relative mRNA of CBX1 (H) and protein levels of CBX1 and α-Tubulin (I) in SUNE1, HONE1 or HK1 cells transfected with siRNA targeting NC (siNC) or CBX1 (siCBX1-1 or -2). Mean (n = 3) ± s.d. One-way ANOVA, *** $P < 0.001$. J, K) Representative images and quantification of colony formation assays (J) and CCK-8 assays (K) in SUNE1 and HONE1 cells transfected with siRNA targeting NC (siNC) or *CBX1* (si*CBX1*-1 or -2). Mean (n = 3 in J, n = 6 in K) ± s.d. ANOVA in J, two-way ANOVA in K, ** $P < 0.01$, *** $P < 0.001$. L) Strategy and amplicon sequencing of CRISPR/Cas9-mediated editing of *CBX1* gene in SUNE1 and HONE1 cells. M) Relative mRNA levels of Her2 in SUNE1 and HONE1 cells stably transfected with sgRNA targeting NC (sgNC) or CBX1 (sg*CBX1*-1 or -2). Mean (n = 3) ± s.d. One-way ANOVA, ns, no significant, *** $P < 0.001$. N) Correlation analysis of average *CBX1* expression in NPC cells and proportion of exhausted $CD8^+$ T cells in all $CD8^+$ T cells (GSE150430 dataset). Pearson R statistical test was used.

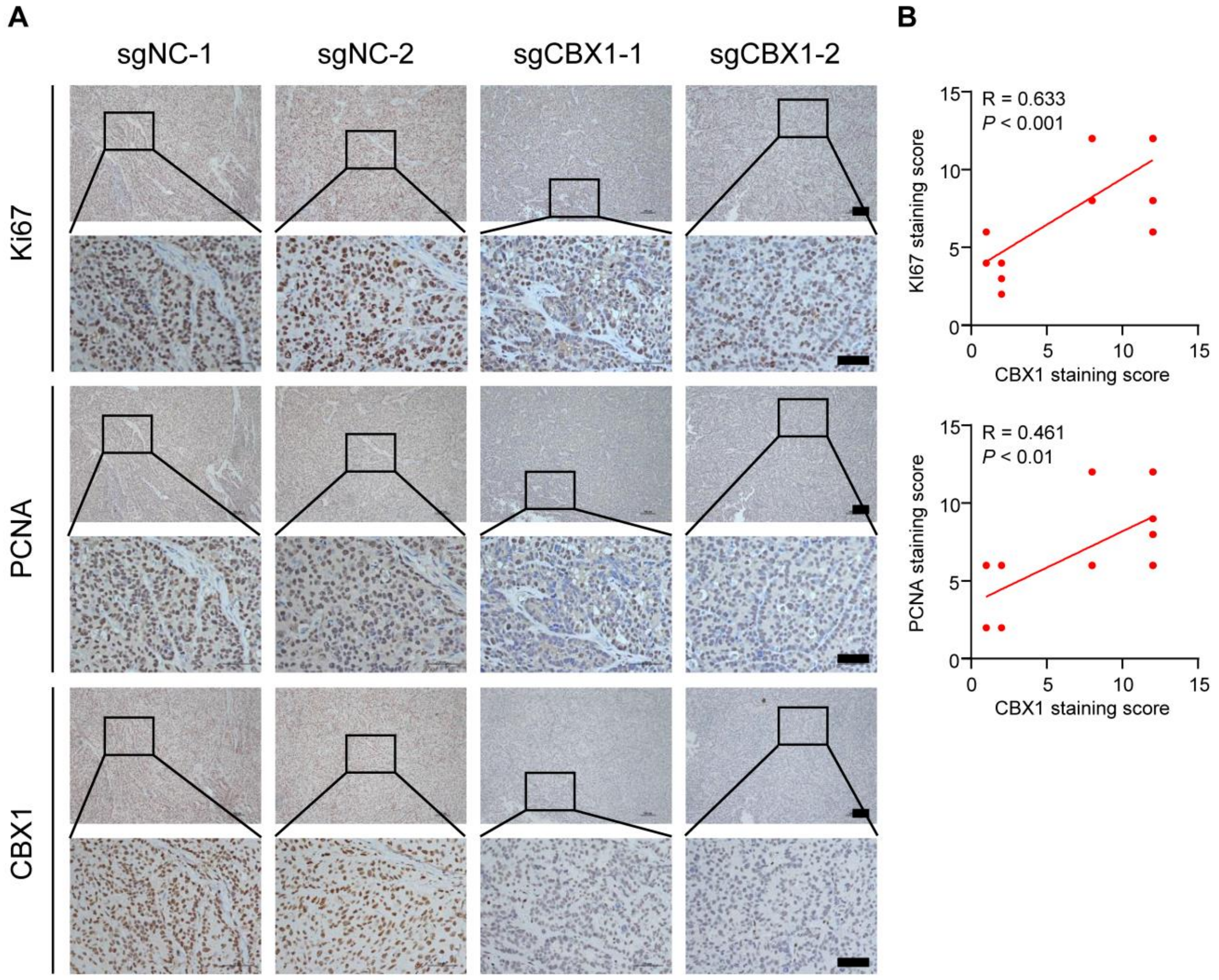

Figure S3. CBX1-knockout group had lower expression of Ki67 and PCNA. A, B) Representative images of IHC staining anti-CBX1, anti-Ki67 or anti-PCNA (A) and correlation analysis of CBX1 expression and Ki67 or PCNA expression according to IHC score statistic (B) in the axilla tumors of nude mice from sgNC or sgCBX1 group. Scale bars, 50 μm. Pearson R statistical test was used.

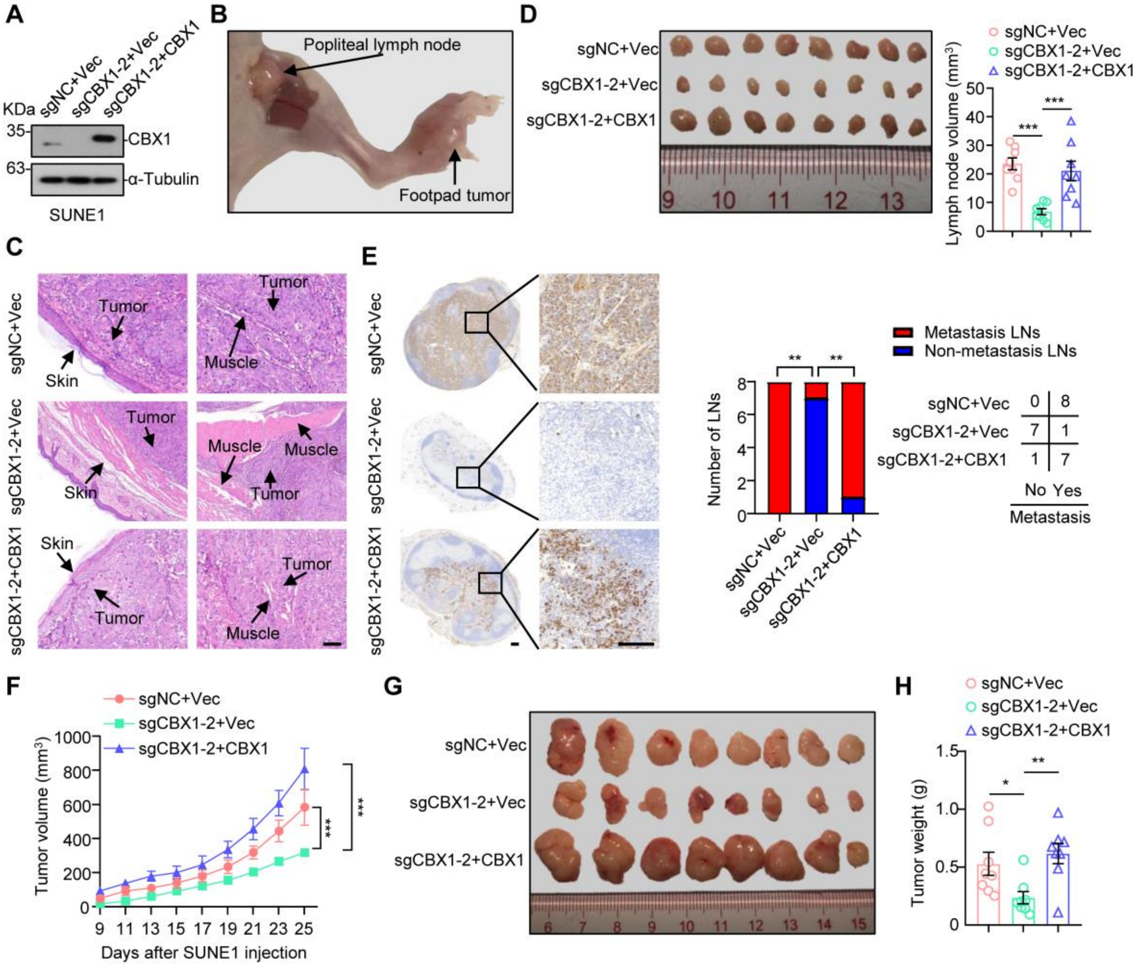

Figure S4. Restoring the expression of CBX1 significantly rescued the inhibitory effect of CBX1 knockout on NPC cell metastasis and tumor growth. A) Western blotting of CBX1 and α-tubulin in SUNE1-sgNC cells with stable overexpression of empty vector, and SUNE1-sgCBX1-2 cells with stable overexpression of empty vector or FLAG-CBX1. B-E) The indicated cells were injected into footpads of nude mice to construct an inguinal lymph node metastasis model. Representative images of primary foot pad tumor and metastatic inguinal lymph node (B) and the primary tumors in footpads following H&E staining (C); images and quantification of the volumes of the inguinal lymph nodes (D); representative images of IHC staining (anti-keratin) of the inguinal lymph nodes; number of metastasis and non-metastasis inguinal lymph nodes (E). F-H) The indicated cells were injected into the axilla of nude mice to construct tumor growth model. Tumor growth curves (F), images (G) and weight (H) of tumors. Mean (n = 8) ± s.d; One-way ANOVA in (D, H), chi-square (χ2) test in (E), two-way ANOVA in F, * $P < 0.05$, ** $P < 0.01$, *** $P < 0.001$; Scale bars, 100 μm.

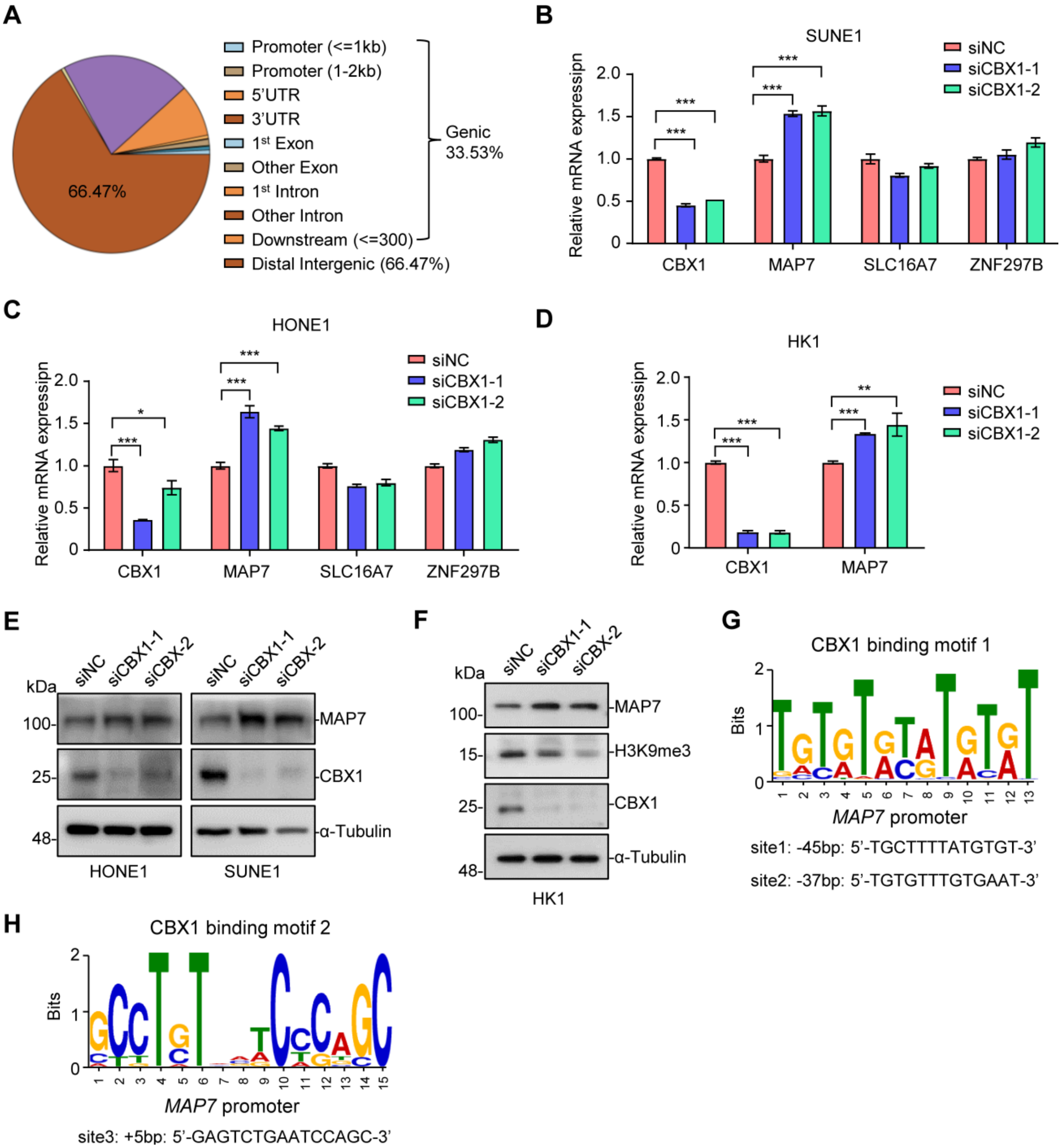

Figure S5. CBX1 targets MAP7 for H3K9me3-mediated transcriptional repression. A) ChIP-seq analysis in SUNE1 cells that overexpressed FLAG-CBX1. Distribution of genes enriched by FLAG-CBX1 (q value < 0.05). B, C) Relative mRNA levels of CBX1, MAP7, SLC16A7 and ZNF297B in SUNE1 (B) and HONE1 (C) cells transfected with siRNA targeting NC (siNC) or CBX1 (siCBX1-1 or -2). Mean (n = 3) ± s.d. One-way ANOVA, * $P < 0.05$, *** $P < 0.001$. D) Relative mRNA levels of CBX1 and MAP7 in HK1 cells transfected with siRNA targeting NC

(siNC) or CBX1 (siCBX1-1 or -2). Mean (n = 3) ± s.d; One-way ANOVA, ** $P < 0.01$, *** $P < 0.001$. E) Western blotting of MAP7, CBX1 and α-Tubulin in SUNE1 and HONE1 cells transfected with siRNA targeting NC (siNC) or CBX1 (siCBX1-1 or -2). F) Western blotting of MAP7, H3K9me3, CBX1 and α-Tubulin in HK1 cells transfected with siRNA targeting NC (siNC) or CBX1 (siCBX1-1 or 2). G, H) CBX1 binding motifs and CBX1 binding sites on MAP7 promoter.

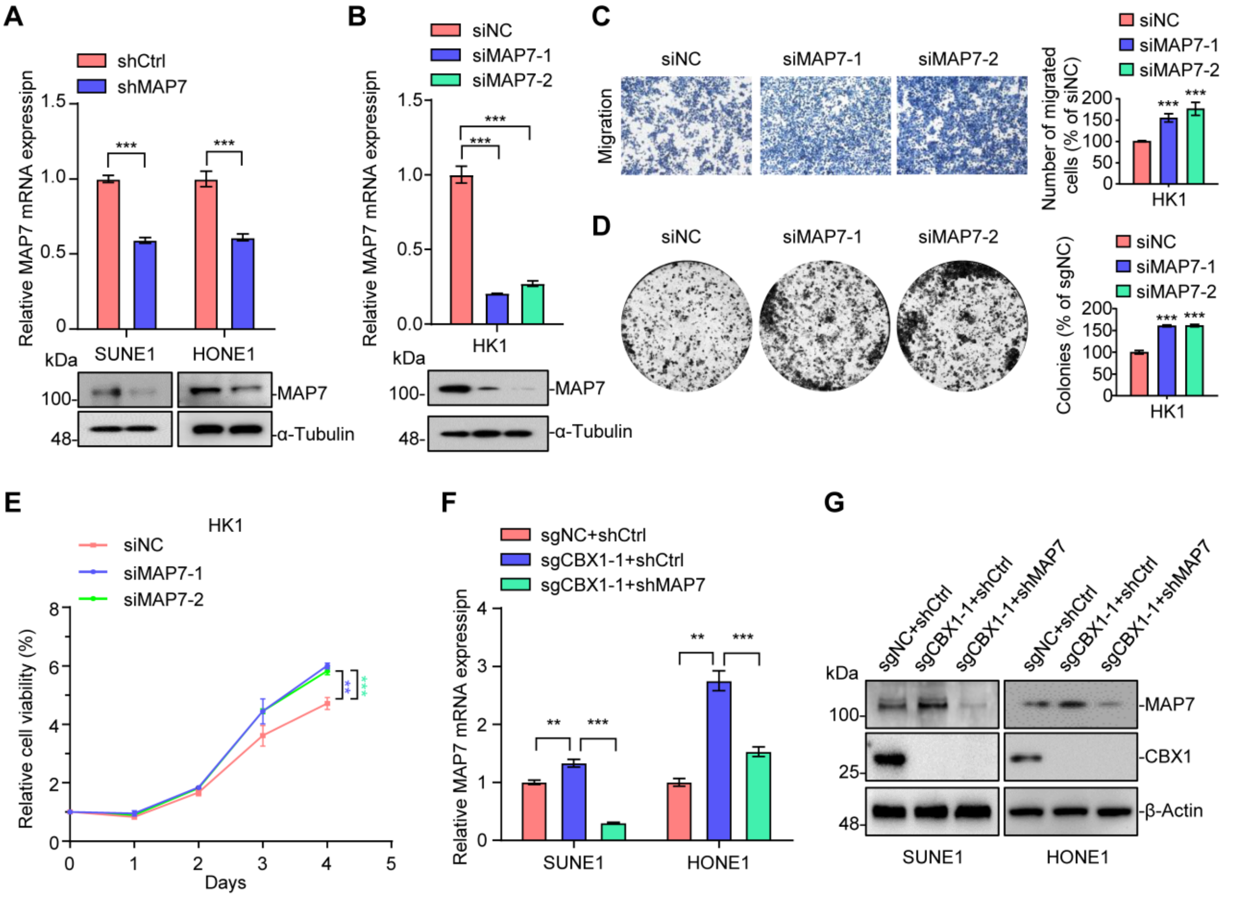

Figure S6. Knockdown of MAP7 significantly promoted NPC cell migration and proliferation. A) Relative mRNA of MAP7 (top) and protein levels of MAP7 and α-Tubulin (bottom) in SUNE1 and HONE1 cells transfected with shRNA targeting control (shCtrl) or MAP7 (shMAP7). Mean (n = 3) ± s.d. Student's *t*-test, *** $P < 0.001$. B) Relative mRNA level of MAP7 (top) and protein levels of MAP7 and α-Tubulin (bottom) in HK1 cells transfected with siRNA targeting control (siNC) or MAP7 (siMAP7-1 or -2). Mean (n = 3) ± s.d. One-way ANOVA, *** $P < 0.001$. C-E) Representative images and quantification of transwell assay (C, without Matrigel), colony formation assays (D) and CCK-8 assays (E) in HK1 cells that transfected with siRNA targeting control (siNC) or MAP7 (siMAP7-1 or -2). Mean (n = 5 in C, n = 3 in D and n = 6 in E) ± s.d; One-way ANOVA in C and D, two-way ANOVA in E, ** $P < 0.01$. *** $P < 0.001$. F, G) Relative mRNA of MAP7 (F) and protein levels of MAP7 and α-Tubulin (G) in SUNE1 and HONE1 cells expressing sgNC or sgCBX1 transfected with shRNA targeting control (shCtrl) or MAP7 (shMAP7). Mean (n = 3) ± s.d. One-way ANOVA, ** $P < 0.01$, *** $P < 0.001$.

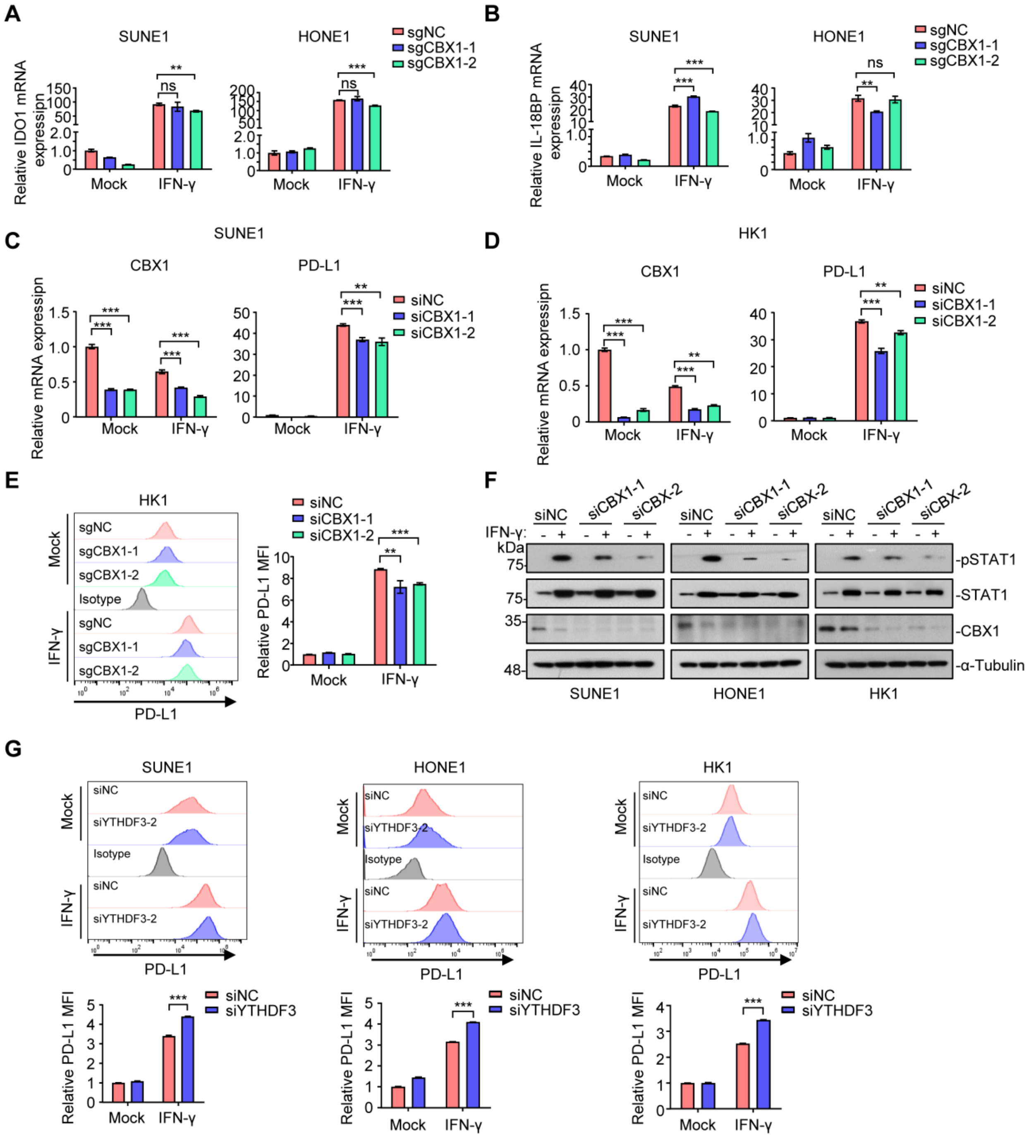

Figure S7. CBX1 inhibition suppresses PD-L1 expression-induced by IFN-γ-STAT1 signaling. A, B) Relative mRNA of IDO1 and IL-18BP in SUNE1 and HONE1 cells stably transfected with sgRNA targeting NC (sgNC) or CBX1 (sgCBX1-1 or -2) treated with or without IFN-γ for 24 hours (10 ng/ml). C, D) Relative mRNA of CBX1 and PD-L1 in SUNE1 (C) or HK1 (D) cells transfected with siRNA targeting NC (siNC) or CBX1 (siCBX1-1 or -2) treated with or without

IFN-γ for 24 hours (10 ng/ml). Mean (n = 3) ± s.d. One-way ANOVA, ** $P < 0.01$, *** $P < 0.001$, ns, no significant. E) Flow cytometry analysis of PD-L1 expression in siNC, siCBX1-1 or siCBX1-2 HK1 cells treated with or without IFN-γ for 24 hours (10 ng/ml). MFI, mean fluorescent intensity. Mean (n = 3) ± s.d. One-way ANOVA, ** $P < 0.01$.*** $P < 0.001$. F) Western blotting of pSTAT1, STAT1, FLAG-CBX1 and α-Tubulin in SUNE1 and HONE1 cells transfected with siRNA targeting NC (siNC) or CBX1 (siCBX1-1 or -2) treated with or without IFN-γ for 24 hours (10 ng/ml). G) Flow cytometry analyzing of PD-L1 expression in siNC or siYTHDF3 SUNE1, HONE1 or HK1 cells treated with or without IFN-γ for 24 hours (10 ng/ml). MFI, mean fluorescent intensity. Mean (n = 3) ± s.d. One-way ANOVA, *** $P < 0.001$.

Table S1. Writers, erasers and readers involved in histone methylation that identified from the WERAM database (http://weram.biocuckoo.org/).

Histone methyltransferase (Writers)	Histone demethylase (Erasers)	Histone methylation reader (Readers)		
ASH1L	AL513412.1	AF10	JADE3	SCML2
DOT1L	HR	AIRE	KAT6B	SFMBT1
EHMT1	JMJD1C	ANK2	L3MBTL1	SFMBT2
EHMT2	JMJD6	ATRX	L3MBTL2	Sgf29
EZH1	KDM1A	BAZ2A	L3MBTL3	SMN
EZH2	KDM2A	BPTF	L3MBTL4	SMN1
KMT2A	KDM2B	BRPF1	LRWD1	SMN2
KMT2B	KDM3A	CBX1	MBTD1	SMNDC1
KMT2C	KDM3B	CBX2	MLLT6	SND1
KMT2D	KDM4A	CBX3	MORC3	SPIN2A
KMT2E	KDM4B	CBX4	MORC4	SPIN2B
MECOM	KDM4C	CBX5	MORF4L2	SPIN3
NSD1	KDM4D	CBX6	MPP8	SPIN4
NSD2	KDM4E	CBX7	MRG15	Spindlin1
NSD3	KDM5A	CBX8	MSH6	TAF3
PRDM1	KDM5B	CDY1	MSL3	TCF19
PRDM12	KDM5C	CDY1B	MTF2	TDRD1
PRDM16	KDM5D	CDY2A	NCAPD3	TDRD15
PRDM2	KDM6A	CDY2B	NCAPG2	TDRD3
PRDM5	KDM6B	CDYL	N-PAC	TDRD6
PRDM7	KDM6C	CDYL2	ORC1	TP53BP1
PRDM8	KDM7A	CHD1	PHF1	TRIM24
PRDM9	KDM8	CHD2	PHF10	UHRF1
PRMT1	MINA	CHD3	PHF12	UHRF2
PRMT4	NO66	CHD4	PHF13	WDR5
PRMT5	PHF2	CHD5	PHF14	HDGFL1
PRMT6	PHF8	CXXC1	PHF19	ING1
PRMT8	RP11-146B14.1	DIDO1	PHF23	ING2
SETD1A		DNMT3A	PHF3	ING3
SETD1B		DNMT3B	PHRF1	ING4
SETD2		DNMT3L	PSIP1	PHF20
SETD6		DPF1	Pygo1	PHF20L1
SETD7		DPF2	Pygo2	PHF21A
SETD8		EED	RAG2	PHF21B
SETDB1		HDGF	RNF17	ZCWPW1
SETDB2		HDGF2	RP11-382A20.3	ZCWPW2
SETMAR		ING5	RSF1	ZMYND11
SMYD1		JADE1	SCMH1	
SMYD2		JADE2	WDR5B	
SMYD3				
SUV39H1				

SUV39H2
SUV420H1
SUV420H2

Table S2. Correlations between CBX1 expression levels and clinical features in nasopharyngeal carcinoma patients.

**Characteristic**	**Low expression group (%)** n = 138	**High expression group (%)** n = 66	***P*-value***
Age			0.6553
≤45	66 (47.83)	34 (51.52)	
>45	72 (52.17)	32 (48.48)	
Gender			0.2317
Male	107 (77.54)	46 (69.70)	
Female	31 (22.46)	20 (30.30)	
TNM Stage†			0.3089
II	40 (28.99)	14 (21.21)	
III/IV	98 (71.01)	52 (78.79)	
Distant metastasis			**<0.001**
Yes	12 (8.70)	19 (28.79)	
No	126 (91.30)	47 (71.21)	
Disease			**<0.001**
Yes	25 (18.12)	30 (45.45)	
No	113 (81.88)	36 (54.55)	
Death			**<0.001**
Yes	17 (12.32)	28 (42.42)	
No	121 (87.68)	38 (57.58)	

* Chi-square test.

† All patients were restaged according to the 7th edition of the AJCC Cancer Staging Manual.

Table S3. List of primers used in this study.

Gene	Sequence (5’ to 3’)
**Real time RT-PCR primers**	
CBX1-F	GAGCTACAGACTCCAGTGGAGA
CBX1-R	GTAGGAATGCCACGTCAGCCTT
MAP7-F	GTACTCTTCCTCACATCTGGCAC
MAP7-R	GCCAGGCAAATGAGGAAGAGAC
YTHDF1-F	CAAGCACACAACCTCCATCTTCG
YTHDF1-R	GTAAGAAACTGGTTCGCCCTCAT
YTHDF2-F	TAGCCAGCTACAAGCACACCAC
YTHDF2-R	CAACCGTTGCTGCAGTCTGTGT
YTHDF3-F	GCTACTTTCAAGCATACCACCTC
YTHDF3-R	ACAGGACATCTTCATACGGTTATTG
PD-L1-F	TGCCGACTACAAGCGAATTACTG
PD-L1-R	CTGCTTGTCCAGATGACTTCGG
IDO1-F	GCCTGATCTCATAGAGTCTGGC
IDO1-R	TGCATCCCAGAACTAGACGTGC
IL-18BP-F	GTGTCCAGCATTGGAAGTGACC
IL-18BP-R	GGAGGTGCTCAATGAAGGAACC
Her2-F	GGAAGTACACGATGCGGAGACT
Her2-R	ACCTTCCTCAGCTCCGTCTCTT
SLC16A7-F	TGCTGGCTGTTATGTACGCAGG
SLC16A7-R	GCCAACACCATTCCAAGACAGC
ZNF297B-F	TGAGCACGGAAATGGCAAGCCA
ZNF297B-R	GATCCAGCGTTTGTGAGCCATG
GAPDH-F	GTCTCCTCTGACTTCAACAGCG
GAPDH-R	ACCACCCTGTTGCTGTAGCCAA
**shRNA primers**	
shRNA-*YTHDF1*-#1-F	CCGGGTTCGTTACATCAGAAGGATACTCGAGTATCCTTCTGATGTAACGAACTTTTTG
shRNA-*YTHDF1*-#1-R	AATTCAAAAAGTTCGTTACATCAGAAGGATACTCGAGTATCCTTCTGATGTAACGAAC
shRNA-*YTHDF1*-#2-F	CCGGGCCGTCCATTGGATTTCCTTACTCGAGTAAGGAAATCCAATGGACGGCTTTTTG
shRNA-*YTHDF1*-#2-R	AATTCAAAAAGCCGTCCATTGGATTTCCTTACTCGAGTAAGGAAATCCAATGGACGGC
shRNA-*YTHDF2*-#1-F	CCGGCTAGAGAACAACGAGAATAAACTCGAGTTTATTCTCGTTGTTCTCTAGTTTTTG
shRNA-*YTHDF2*-#1-R	AATTCAAAAACTAGAGAACAACGAGAATAAACTCGAGTTTATTCTCGTTGTTCTCTAG
shRNA-*YTHDF2*-#2-F	CCGGGCAGACTTGCAGTTTAAGTATCTCGAGATACTTAAACTGCAAGTCTGCTTTTTG
shRNA-*YTHDF2*-#2-R	AATTCAAAAAGCAGACTTGCAGTTTAAGTATCTCGAGATACTTAAACTGCAAGTCTGC

shRNA-*MAP7*-F	CCGGGCCCTCTTACATAATGTATTTCTCGAGAAATACAT TATGTAAGAGGGCTTTTTG
shRNA-*MAP7*-R	AATTCAAAAAGCCCTCTTACATAATGTATTTCTCGAGA AATACATTATGTAAGAGGGC
**siRNA sequences**	
siRNA-*YTHDF3*-#1	GACTAGCATTGCAACCAAT
siRNA-*YTHDF3*-#2	GGACAATCAACACAAAGT
siRNA-CBX1-1	GTGGAGAGCTCATGTTCCT
siRNA-CBX1-2	GCGCAAAGCTGATTCTGAT
siRNA-*MAP7*-1	GCAGCGTTGTTAACAGACT
siRNA-*MAP7*-2	CACCTTTAGTGAAGGTAGA
**sgRNA sequences**	
sgRNA-human-*CBX1*-1	TTACAGTCAGAAAAGCCACG
sgRNA-human-*CBX1*-2	GTGGCATTCCTACCCCTCGG
sgRNA-human-*CBX1*-3	AAAAGTTCTCGACCGTCGAG
sgRNA-mouse-*Cbx1*-1	TTACAGTCAGAAAAGCCACG
sgRNA-mouse-*Cbx1*-2	AAACCCCGGGCAAAGCCTCG
**ChIP-qPCR primers**	
MAP7-F	TAGCACTTACACTCCAGACGG
MAP7-R	TGAGTTGTCACCAAAGCCCA
**m^6A-RIP-qPCR/RIP-qPCR primers**	
CBX1-site1/2-F	TTGCTGCGAAAAGCAAAGGG
CBX1-site1/2-R	CTGTGGGTTGTGGAGATGCT
CBX1-site3/4-F	TAAGCCAAGGTGTTCCCTGC
CBX1-site3/4-R	AGACCTTCCCTCCTCGTCAA
